# Exploration on the effect of anserine on the alleviation of DVT and its molecular mechanism

**DOI:** 10.3389/fphar.2024.1402758

**Published:** 2024-05-23

**Authors:** Yan Li, Jingping Ge, Yuanyuan Yin, Juan He, Longcheng Shang

**Affiliations:** ^1^ Department of Vascular and Interventional Radiology, Nanjing First Hospital, Nanjing Medical University, Nanjing, Jiangsu, China; ^2^ Department of General Surgery, Nanjing First Hospital, Nanjing Medical University, Nanjing, Jiangsu, China

**Keywords:** deep vein thrombosis, anserine, multiomics techniques, non-target metabolomics, MYB, CARNMT1

## Abstract

**Background:**

This study aimed to explore the regulatory effect of anserine on HUVEC cell injury and thrombosis in deep venous thrombosis (DVT) rats, and to elucidate the underlying molecular mechanisms.

**Methods:**

Non-targeted metabolomics data analyses were conducted using an ultra-performance liquid chromatography system Vanquish UHPLC and mass spectrometer to detect plasma metabolism profiles. The transcriptome sequencing and gene intervention experiments were performed to verify the regulatory effect. Further *in vivo* and *in vitro* experiments were performed. Enzyme-linked immunosorbent assay was used to detect the levels of P-selectin, E-selectin, and vWF, hematoxylin-eosin (HE) staining was performed to observe thrombotic and inflammatory cell infiltration, flow cytometry and TUNEL assays were performed to detect apoptosis, and qPCR and WB assays were conducted to determine the gene and protein expression.

**Results:**

Anserine alleviated HUVECs injury, reduced adhesion molecule expression, and inflammation. It decreased P-selectin, E-selectin, vWF, THBD, TFPI levels, and apoptosis while promoting NOS3, ET-1, and NO release in HUVECs. In DVT rats, anserine reduced P-selectin, E-selectin, vWF, thrombosis, cell infiltration, apoptosis, and promoted NO release. Transcriptome sequencing and gene intervention confirmed anserine’s regulation of the PI3K-Akt pathway and coagulation via MYB. CARNMT1, a regulatory enzyme for anserine metabolism, increased anserine content, inhibiting coagulation, thrombosis, cell infiltration, and promoting NO release in rats.

**Conclusion:**

This study confirmed anserine could alleviate DVT by improving the inflammatory response, inhibiting blood agglutination, and promoting vasodilation, providing new potential therapeutic targets, important scientific evidence for the development of DVT management, and new clues for an in-depth understanding of its molecular mechanisms.

## 1 Introduction

Deep venous thrombosis (DVT) is characterized by abnormal coagulation of blood in the deep vein, resulting in venous reflux obstructive disease, which often occurs in the lower limbs. The main causes of venous thrombosis are slow blood flow, damage to the vein wall, hypercoagulability with typical maniffestations (Akgül et al., 2016; Imran et al., 2018) swelling and pain in the lower limbs. If not treated promptly, the thrombus spreads to the deep vein trunk of the limbs. Acute embolus detachment is more likely to cause pulmonary embolism, which seriously affects the life and health of patients (Di Nisio et al., 2016; de Athayde Soares et al., 2019).

Studies have shown that the main reasons for the occurrence of lower-extremity DVT include hypercoagulation, intravenous injury, and slow blood flow, which are more common in patients with a history of major surgery, severe trauma, long-term bedridden status, limb immobilization, and malignant tumor diseases (Kim et al., 2021). Venous stagnation, venous wall injury, and blood hypercoagulation may be the main causes of DVT in the lower extremity. Old age, pregnancy, long-term smoking history, and obesity are also closely associated with DVT in the lower extremities (Myers, 2015; Di Nisio et al., 2016). Surgery or trauma leads to local vascular contusion in the body, which can cause vascular endometrial damage to the patient. DVT may develop under the combined action of endothelin and inflammatory factors. During the operation, due to factors such as cutting blood vessels and anesthesia, the possibility of DVT in patients is also increased (Rabinovich et al., 2015).

DVT has been shown to be closely associated with inflammatory response. After the body is injured, inflammatory factors accumulate, accelerating the hypercoagulable state of the body, causing adverse consequences such as platelet aggregation, endothelial injury, and thrombosis (Halici et al., 2014; Brandt et al., 2018). Thrombotic therapy includes both nonsurgical and surgical thrombectomies. Nonsurgical treatments include thrombolytic therapy, anticoagulant therapy, and deaggregation therapy (Zhang et al., 2021). However, thrombolytic therapy is associated with an increased risk of hemorrhea and does not show a mortality benefit in patients with DVT (Guyatt et al., 2012). Anticoagulant therapy is an important method for preventing DVT, and the main prophylactic drugs used are common heparin, low-molecular-weight heparin, X a factor inhibitory engraving, and vitamin K antagonists. Surgical thrombectomy carries the risks of surgical bleeding, infection, and anesthesia, especially in patients with other underlying conditions or comorbidities (Kakkos et al., 2021). Postoperative complications are also a potential risk for surgical thrombectomy, and although surgical treatment can remove DVT, it cannot solve the problem of patients being unable to prevent new thrombosis due to long-term bed bedridden, tumors, and other potential causes in the body. Therefore, it is of great significance to explore new drugs for DVT treatment and their corresponding regulatory mechanisms to provide a reference for the effective treatment of DVT.

Metabolomics is often used to study potential metabolic pathways, biomarkers, and therapeutic targets of diseases, and functional metabolomics is increasingly used to study systemic effects on hosts by identifying metabolites with specific functions (Yan and Xu, 2018; Liu and Ren, 2024). Examples of applied metabolomics have been found to identify disease biomarkers and therapeutic targets but are rarely reported in DVT. The present protocol used non-target metabolomics to study the differences in blood metabolites between DVT rat models and normal rats, screening potential metabolites that could alleviate DVT, providing new therapeutic targets for disease management, and combining transcriptome sequencing technology to study the molecular mechanism of potential metabolites to alleviate endothelial function damage.

## 2 Materials and methods

### 2.1 Constructing a DVT rat model

Sixty male Sprague-Dawley (SD) rats, SPF grade, aged 2 months, weighing 280–300 g were purchased from Chongqing Ensiweier Biotechnology Co., Ltd. Twelve rats were divided into a sham group and a DVT model group using a random number table simple randomization method, six in each group. All rats were fed with food and drinking water freely under the environmental conditions on alternate days and nights for 12 h, constant humidity at 20°C–25°C. Adaptive feeding was terminated after 1 week for subsequent experiments. DVT rat models were induced by ligation of the inferior vena cava. The animals were fasted for 12 h before surgery, but provided drinking water. The weights of the rats were then recorded. Subsequently, the rats were intraperitoneally administered 30 mg/kg of 0.3% pentobarbital sodium (Beijing Zhongheng Science Company, P3761) for anesthesia. The rats were fixed to a bench in a supine position with prepared skin, disinfected in the middle abdominal surgical area, and cut open along the white abdominal line. The small intestine was wrapped with a piece of wet gauze and placed in the right abdominal cavity of the animal to expose the inferior vena cava and its main branches. Ligation of the inferior vena cava and its main branches was performed using a 6-0 nylon suture approximately 1 cm below the left renal vein. Normal saline containing penicillin (McLean, G815743-5G) was sprayed before the abdominal cavity was closed to establish a rat model of DVT. The sham group underwent the same procedure as the model group, except that no veins were ligated. After 24 h of modeling, the rats were anesthetized and the abdominal sutures were removed (the inferior vena cava and its main branch lumen were observed to be thickened, the color was purple-black, and solid substances were formed, indicating that the modeling of DVT rats was successful). Blood from each group was collected and centrifuged to separate the serum. After the serum was aspirated, and the liquid nitrogen was used for quick freezing. Sample storage condition was set at −80°C. All animals received care in compliance with the American, European, or any other Convention on Animal Care, with the comment that the study was approved by Experimental Animal Ethics Committee of Nanjing First Hospital, Nanjing Medical University (approval no. DWSY-20210812).

### 2.2 Non-target metabolomics analysis

The serum of the rat model was tested for nontarget metabolomics. The groups included a sham group (15 rats) and a DVT model group (15 rats). Non-targeted metabolomic data analyses were performed using an ultra-performance liquid chromatography system (Vanquish UHPLC, Germany) and a Q Exactive™ HF mass spectrometer (Germany). Raw data were imported into the Compound Discoverer 3.1 software for peak alignment, extraction, and identification based on retention time, M/Z ratio, and ion area. For the multivariate statistical analysis, we employed the metaX software for data processing purposes, which facilitated the conversion of data and conducted principal component analysis (PCA) and partial least squares discriminant analysis (PLS-DA), allowing to the acquisition of VIP values for each metabolite. Fold-changes in metabolites between both groups were computed as FC values. Default criteria for differential metabolite screening were VIP > 1, *p* < 0.05, and FC ≥ 2 or ≤ 0.5. Metabolite identification and annotation were performed employing KEGG database (https://www.genome.jp/kegg/pathway.html), HMDB database (https://hmdb.ca/metabolites), and LIPIDMaps database (http://www.lipidmaps.org/).

Volcano map was plotted using R package ggplot2, combining VIP values of the metabolite, log_2_ (FC), and -log10 (*p*-value), for the screening of metabolite of interest. Meanwhile, the metabolite data normalization was conducted based on z-scores. Bubble maps were plotted using the R package ggplot2; Metabolic functions and pathways were analyzed using the KEGG database. A metabolic pathway was deemed enriched when the ratio of x/n to y/n exceeded a certain threshold. Furthermore, if the *p*-values associated with the metabolic pathway were less than 0.05, it was considered significantly enriched.

### 2.3 Culture and treatment of human umbilical vein endothelial cells (HUVECs)

HUVECs (Procell Life Science&Technology Co., Ltd.) (Item No.: CL-0122) were obtained, prepared in a Dulbecco’s modified Eagle’s medium (DMEM)/F12 medium (Gibco, United States, C11330500BT) containing 1% penicillin/streptomycin solution (Beyotime, China, C0222) and 10% FBS (Life-iLab, China, AC03L055), and placed in incubators at 37°C and 5% CO_2_. Cell growth was observed, and when confluence reached more than 80%, well-grown HUVECs were selected for passages at 1:3. The cells were digested with an appropriate quantity of 0.25% pancreatin solution and adjusted to an appropriate concentration of 1 × 10^5^/mL. HUVECs were seeded in 96-well plates (100 μL/well) and incubated in an incubator for 24 h. Based on the operation procedures, the plates were prepared and added with 500 μL of the solution, 2 mL to the 6-well plates, 3–4 mL to the T25 flask for culture.

To detect the effect of anserine (Yuanye Biotechnology, China, S27475-250 mg) on endothelial cell injury, cells in the model and anserine groups were treated with TNF-α (10 μg/L, GenScript, China, Z01001) for 24 h to induce endothelial cell injury. The anserine treatment groups were treated with two doses (50 and 500 μg/mL) for 24 h, and the blank control group was administered normal saline.

### 2.4 CCK-8 detection of cell proliferation

At a cell concentration of 1 × 10^5^/mL, the cells were inoculated into a 96-well plate at 100 μL/well and cultured 24 h. Subsequently, 10 μL of CCK-8 solution (Beyotime, China, C0038) was added to each well, followed by incubation. The absorbance value was then detected at 450 nm using a microplate detector (CMax Plus, United States, Molecular Devices), and the cell proliferative viability was analyzed.

### 2.5 Flow cytometry detection of cell apoptosis

Apoptosis was assessed using flow cytometry, following the manufacturer’s instructions of the Annexin V FITC/PI apoptosis kit (Yeasen Biotechnology (Shanghai) Co., Ltd., 40302ES50). After different treatments in different cell groups, each group was set up with three replicates and centrifuged at 1000 rpm. After removal of supernatant, cells were obtained and resuspended gently in PBS for number calculation. The suspended cells (1 × 10^6^) were centrifuged at 1000 rpm for 3 min; the supernatant was discarded; and 195 μL of Annexin V-FITC-A binding solution was added. Next, 5 μL of AnnexinV-FITC was added, evenly mixed with care for incubation away from light for 10 min. Propidium Iodide (5 μL) was used to fix and stain the cells, CytoFLEX flow cytometry was applied to perform the flow cytometry assays, and the data were processed using FlowJo7.6 software.

### 2.6 ELISA

Each group of cells were cultured and cell supernatant was collected. Concentrations of von Willebrand factor (vWF), P-selectin, and E-selectin were detected using an ELISA kit, according to the manufacturer’s instructions (Wuhan Fine Biotech Co., Ltd., China, EH1064, EH3818, EH0124).

### 2.7 Nitric oxide (NO) level detection

The NO levels in the cells and serum were detected using a NO ELISA kit (SaintBio, BA1460, Shanghai, China).

### 2.8 Gene expression detection

The levels of thrombomodulin (THBD), endothelin 1 (ET-1), tissue factor pathway inhibitor (TFPI), and nitric oxide synthase 3 (NOS3) mRNAs in each group of cells and inferior vena cava tissues of the rats were detected by qPCR assays. The treated cells or tissues were harvested to extract total RNAs using TRIzol. Gel electrophoresis was performed to determine purity, and spectrophotometry (United States, Thermo, NanoDrop One/One C, Thermo Scientific) was used to determine the RNA content. Reverse transcription to cDNA was conducted using reverse transcription kits (Tsingke Biotechnology Co., Ltd., Beijing, TSK302M). Primers were designed according to sequence information based on the NCBI database (Supplementary Table S1), with GAPDH as the internal reference. The effects of THBD, TFPI, ET-1, NOS3, and MYB mRNA expression were detected using RT-qPCR and data analyses were conducted based on a 2^−ΔΔCT^ method.

### 2.9 Protein expression detection

RIPA lysate was utilized to lyse the cells and extract total proteins, and the protein concentrations were determined using the BCA method. A 5 × buffered loading solution and PBS were provided according to the protein content, and the proteins were boiled in a water bath at 100°C for 5 min to denature. After cooling, the sample was subjected to 10% sodium dodecyl sulfate polyacrylamide gel electrophoresis (SDS-PAGE) to isolate the target protein. The isolated protein was then transferred to a polyvinylidene fluoride (PVDF) membrane. The current was adjusted to 300 mA, and the membrane transfer time was determined by the weight of the target protein molecule. After the PVDF membrane was fully immersed in 5% skim milk and sealed for 1 h, it was incubated successively with primary antibodies (THBD, TFPI, ET-1, and NOS3) at 4°C overnight with vibration. Subsequently, the membrane was incubated with the secondary antibody (goat anti-rabbit IgG) at room temperature for 60 min. The membrane was placed in a dark room, mixed with luminescent solutions A and B equally according to the required volume, and added to the front membrane to ensure sufficient contact. The membrane was then placed in a nucleic acid protein gel imager (US, Bio-Rad, universal Hood II) to detect and read the relevant band grayscale values using the ImageJ software. The antibodies utilized are listed in Supplementary Table S2.

### 2.10 Rat blood coagulation function test

The modeling method for DVT rats was the same as that described in Section 2.1. The effect of anserine (Yuanye Biotechnology, China, S27475-250 mg) on the alleviation of DVT formation *in vivo* was studied by intragastric administration to rats. The animals were categorized into four groups: sham group, DVT model group, DVT model + low-dose anserine group, and DVT model + high-dose anserine group, with six rats in each group. Sham and DVT groups of rats received a daily administration of 2 mL of normal saline, and those in the L-anserine and H-anserine groups were given a daily administration of 2 mL solutions containing L-anserine (3 mg/mL) and H-anserin (15 mg/mL). After 1 week of continuous intervention, blood samples were obtained from the intraorbital vein of anesthetized rats to measure four blood coagulation parameters: prothrombin time (PT), thrombin time (TT), activated partial thrombin time (APTT), and fibrinogen (FIB). Blood was obtained from the abdominal aorta, and the serum was separated. The weight, length, and thickness of the thrombi were measured in each group. The thrombus tissues used for pathological experiments were stored at room temperature in 4% paraformaldehyde solution (Aladdin, C104190–500 g).

### 2.11 Hematoxylin-eosin (HE) staining

The inferior vena cava segments of each group were routinely fixed, dehydrated using 75%, 80%, 90%, 95%, and 100% ethanol for 1 h, paraffin-embedded, and prepared into 4-μm-thick sections (United States, Thermo, INESSE E E+). The paraffin sections were rehydrated with gradient ethanol, stained with HE staining (Servicebio, G1004; G1002) for 5 min, dehydrated, and sealed, and the histomorphological differences in the venous segment of local thrombosis in each group were observed under a light microscope (Guangzhou Micro-shot Optical Technology Co., Ltd., Mshot MF53).

### 2.12 TUNEL staining detection of apoptosis

Thrombus tissues were fixed using 4% paraformaldehyde, followed by embedding in paraffin and subsequent sectioning. Subsequently, DAB (SA-HRP) TUNEL Cell Apoptosis Detection Kit (Xavier, G1507) was used for the detection of tissue apoptosis. TUNEL staining was conducted, and after incubation, and the cells were examined under a fluorescence microscope after incubation. Statistical analyses using ImageJ image processing software were performed according to the requirements of the kit.

### 2.13 Transcriptome sequencing analysis

The cell culture and treatment methods for HUVECs were the same as those described in Section 2.3. The experimental cells were categorized into three groups: the control group (Control), model group (Model), and model + 500 μg/mL anserine treatment group (Model + Anserine). Each group of cell samples was subjected to RNA-Seq, and the resulting sequences were analyzed to identify DEGs. GO and KEGG pathway enrichment analyses were performed. Key genes associated with the treatment mechanism were screened in combination with the effect of anserine on endothelial function in cell experiments.

### 2.14 MYB gene interferes with lentiviral packaging

The interfering lentiviral vector of MYB was constructed according to the interfering target site sequence (Supplementary Table S3), which was completed by Biomedicine Biotechnology (China). In short, interfering targets were designed from the transcripts of the gene of interest and primer synthesis was conducted. Single-stranded oligo annealing of MYB formed a double-stranded linkage to the linearized pLVX-shRNA1 vector. After transforming the recombinant plasmid with receptor cells, a resistance culture plate was placed, and the recombinant plasmids were uniformly coated with a coater, cultured invertedly at a constant temperature, and multiple positive transformants in the plate were harvested and delivered for sequencing. The correct monoclonal sequences were selected and verified by sequencing. A large quantity of viral target sequence tool vector plasmids (pLVX-shMYB) and auxiliary packaging plasmids (pLV-Helper1.0 and pLV-Helper2.0) were extracted using a de-endotoxin plasmid extraction kit. Their concentration and purity were determined using an ultraviolet absorption method to ensure that the proposed plasmid DNA at A260/A280 was between 1.8 and 2.0.

The MYB gene interfering with lentiviral plasmids and transfection reagents were mixed evenly with serum-free DMEM, followed by an incubation period of 10 min. The mixture was added dropwise to 293T cells and incubated for 6 h. After culturing under normal conditions for 48 h, the cell culture supernatant was collected. After the first collection of cell supernatant, the DMEM was replaced. After 72 h, the DMEM was collected for the second time and mixed with the first supernatant. Following centrifugal concentration and subpackaging in viral tubes, the mixture was frozen at −80°C until use.

### 2.15 MYB function verification experiment

After HUVECs were subjected to passage culture, the control, NC + model, anserine + NC + model, and anserine + MYB interference + model groups were treated, with the exception of the control group. The HUVECs in the remaining groups were treated with TNF-α (10 μg/L) for 24 h to induce endothelial cell injury. MYB interference/no-load lentiviral infection was performed according to group settings. After 72 h, the groups were subjected to a 24-h treatment of anserine (500 μg/mL), while the blank control group was administered normal saline.

### 2.16 Overexpression of CARNMT1 interferes with adeno-associated viral preparation

Screening of shRNA interference sequences and construction of viral vectors were performed by Biomedicine Biotechnology. The shRNA interference sequence was designed and synthesized according to CARNMT1 (Supplementary Table S4).

The DVT animal modeling method was the same as that used in Experiment 2.1. The synthetic rate-limiting enzyme, CARNMT1, involved in the production of anserine, was determined utilizing the KEGG metabolic pathway resource (http://www.kegg.jp). After CARNMT1 was identified, CARNMT1 overexpression and interference with adeno-associated viruses were induced. The rats in each group were administered tail vein injection according to the experimental groups (Sham group; DVT + NC group; DVT + OE-CARNMT1 group; DVT + IN-CARNMT1 group). Six rats from each group were sacrificed 1 week later, and the rat blood samples and inferior vena cava tissues were collected.

### 2.17 Statistical methods

Performing data preprocessing using DecisionLink V1.0 (Team DC, 2023). Duncan’s *post hoc* test for multiple comparisons was performed using independent samples *t*-test. One-way analysis of variance (ANOVA) was conducted using GraphPad Prism software (version 9.0). The data were presented as “mean ± SD”, *p* < 0.01 demonstrated extremely statistical difference, and *p* < 0.05 demonstrated statistical difference.

## 3 Results

### 3.1 Anserine is identified as a differential metabolite in DVT rats

**FIGURE 1:**
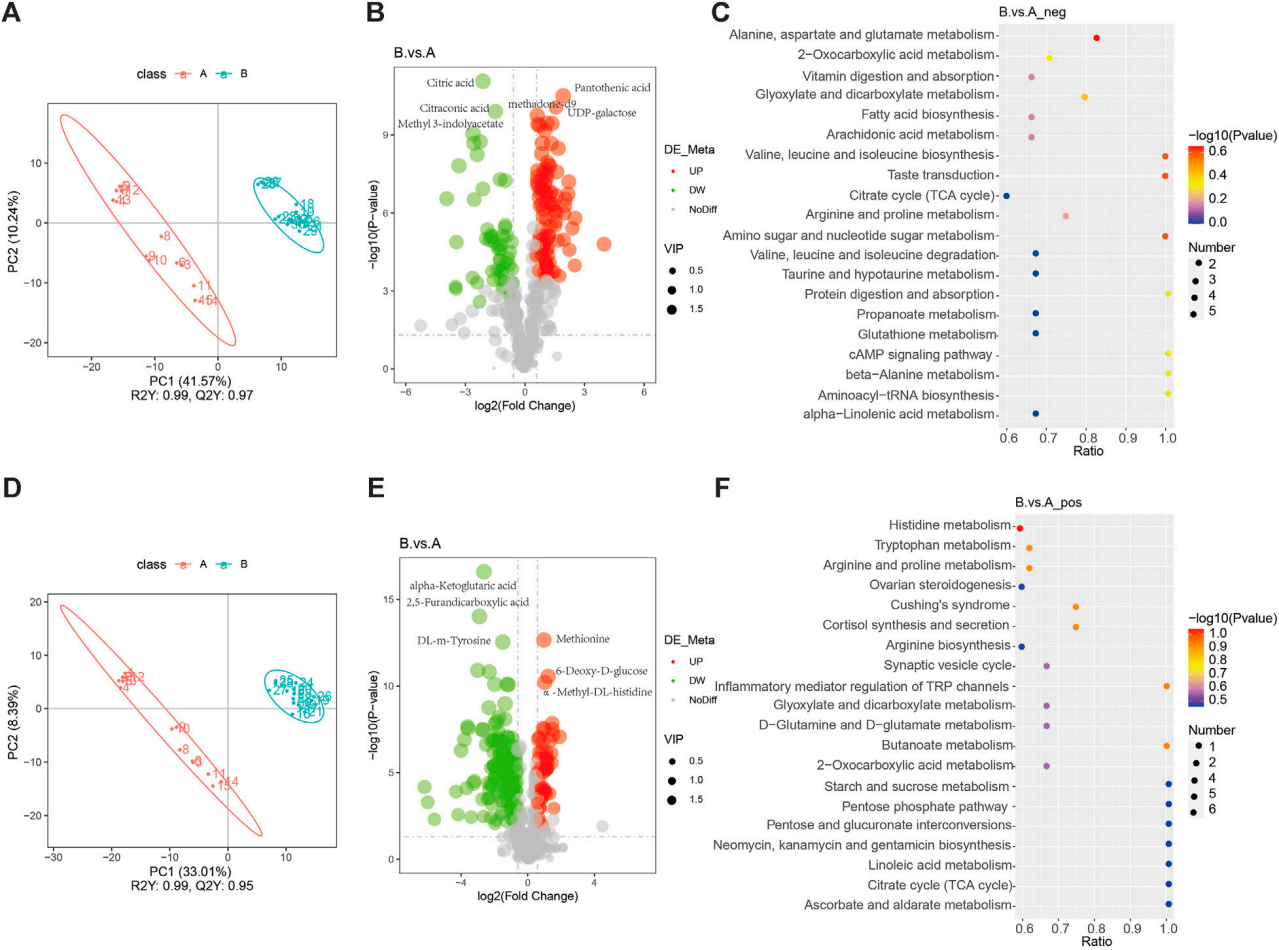
Non-target metabolomics analysis. **(A)** PLS-DA diagram in the anionic mode; **(B)** Volcano map of differential metabolites in the anionic mode; **(C)** Pathways enriched in KEGG analyses of differential metabolites in the anionic mode; **(D)** PLS-DA diagram in the cationic mode; **(E)** Volcano map of differential metabolites in the cationic mode; **(F)** Pathways enriched in KEGG analyses of differential metabolites in the cationic mode.

An orthogonal PLS-DA fraction plot illustrated the separation between the DVT and Sham groups (Figures 1A,D). Based on the criteria of VIP > 1 and *p* < 0.05, A total of 348 metabolites with significant differences were screened between the Sham and DVT groups. Among these metabolites, 54 were found to be upregulated and 138 were downregulated in the cationic mode, while 102 were upregulated and 54 were downregulated in the anionic mode (Supplementary Table S5, Figures 1B,E). Alanine, aspartate, glutamate, and histidine metabolism were the most important biochemical and signal transduction pathways that could be determined by pathway enrichment (Figures 1C,F). Among these differentially expressed metabolites, anserine was found to be enriched in the histidine metabolism pathway. Based on the literature search results, it has been found that serine plays an important physiological role in antioxidant and anti-inflammatory responses (Wu, 2020). This suggests that anserine may have the potential to alleviate DVT and could serve as a promising metabolite for further investigation.

### 3.2 Anserine relieves damage to HUVECs

**FIGURE 2:**
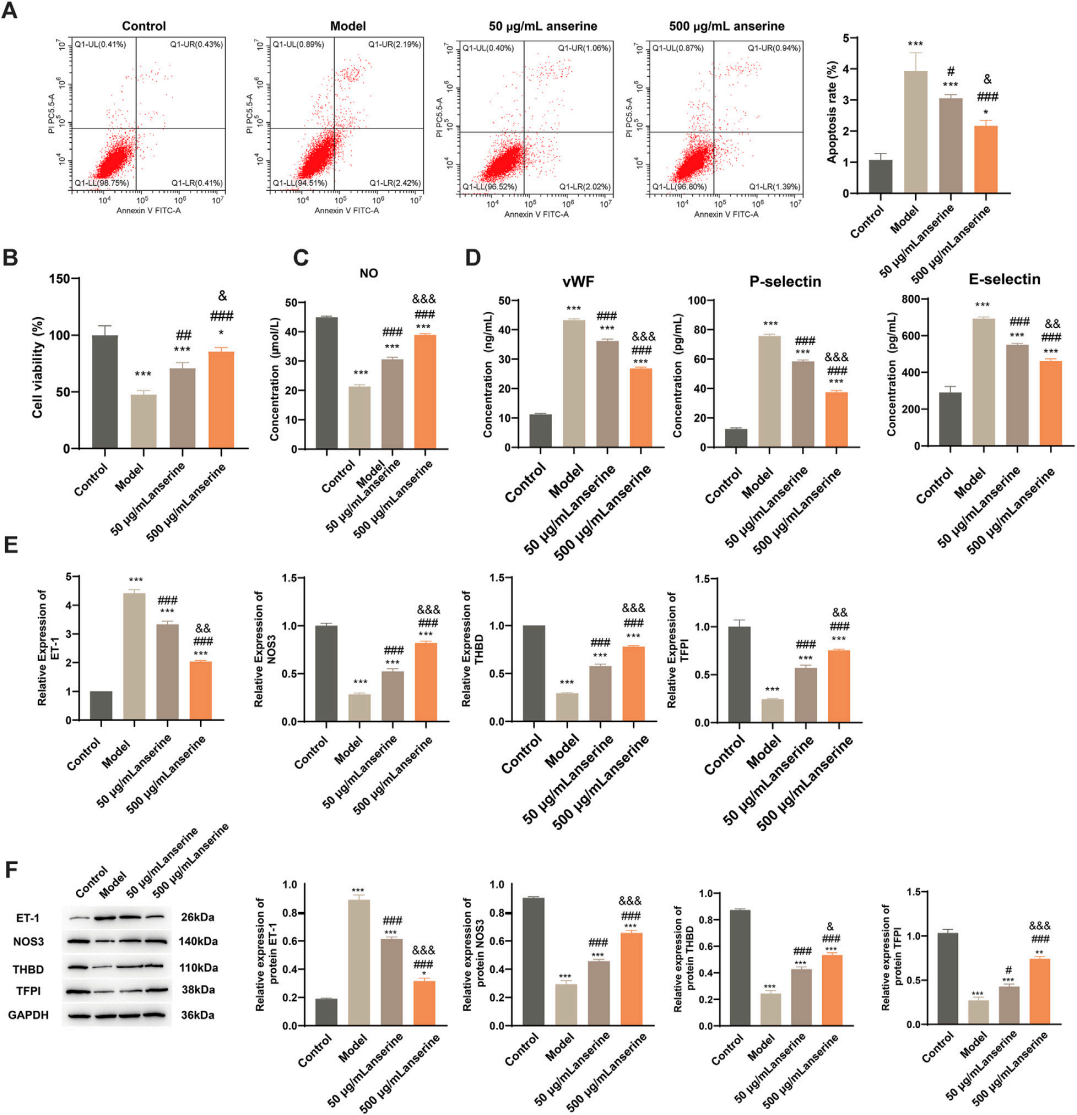
Anserine interventions alleviate HUVECs damage. **(A–E)** After the HUVECs were exposed to TNF-α (10 μg/L) for 24 h, **(A)** apoptosis, **(B)** cell viability, **(C)** cell NO levels, **(D)** cell vWF, P-selectin and E-selectin level was determined by ELISA, **(E)** ET-1, NOS3, THBD and TFPI mRNA, and **(F)** protein expression were detected after intervention with anserine (50 and 500 μg/mL), respectively. Mean ± SD are shown (*n* = 3 in each group). **p < 0.01, ***p < 0.001 compared to the Control group; #*p* < 0.05, ###*p* < 0.001 compared to the Model group; &*p* < 0.05, &&*p* < 0.01, &&&*p* < 0.001 compared to the 50 μg/mL + anserine group.

Studies have shown that the main causes of DVT include blood agglutination and intravenous membrane injury, and are closely related to the inflammatory response. After injury, inflammatory factors aggregate, accelerating the hypercoagulable state of the body, triggering platelet aggregation, endothelial damage, and other adverse consequences, eventually causing thrombosis (Wu and Cheng, 2020). We constructed a model of endothelial injury using TNF-α-treated HUVECs and validated the effects of anserine on endothelial injury, blood agglutination, and inflammatory response of HUVECs at the cellular level using 50 and 500 μg/mL anserine for interventions. Compared with the endothelial injury group, 50 μg/mL and 500 μg/mL anserine-treated cells showed significantly inhibited apoptosis (Figure 2A) and enhanced cell proliferation (Figure 2B). Treatment with 50 and 500 μg/mL anserine promoted NO release from cells (Figure 2C) and inhibited and enhanced the expression of ET-1 and NOS3 genes and proteins, respectively, which might have a positive effect on vasodilation (Figure 2E,F). In addition, 50 μg/mL and 500 μg/mL anserine significantly reduced vWF levels (Figure 2D) and THBD and TFPI gene and protein expression in TNF-α-treated cells (Figures 2E,F). Additionally, cellular levels of P-selectin and E-selectin, which are adhesion molecules involved in the inflammatory response and migration of immune cells, were significantly reduced after anserine treatment (Figure 2D) (Panicker et al., 2017; Purdy et al., 2022).

### 3.3 Anserine inhibits vascular damage and thrombosis in inferior vena cava tissues of DVT rats

**FIGURE 3:**
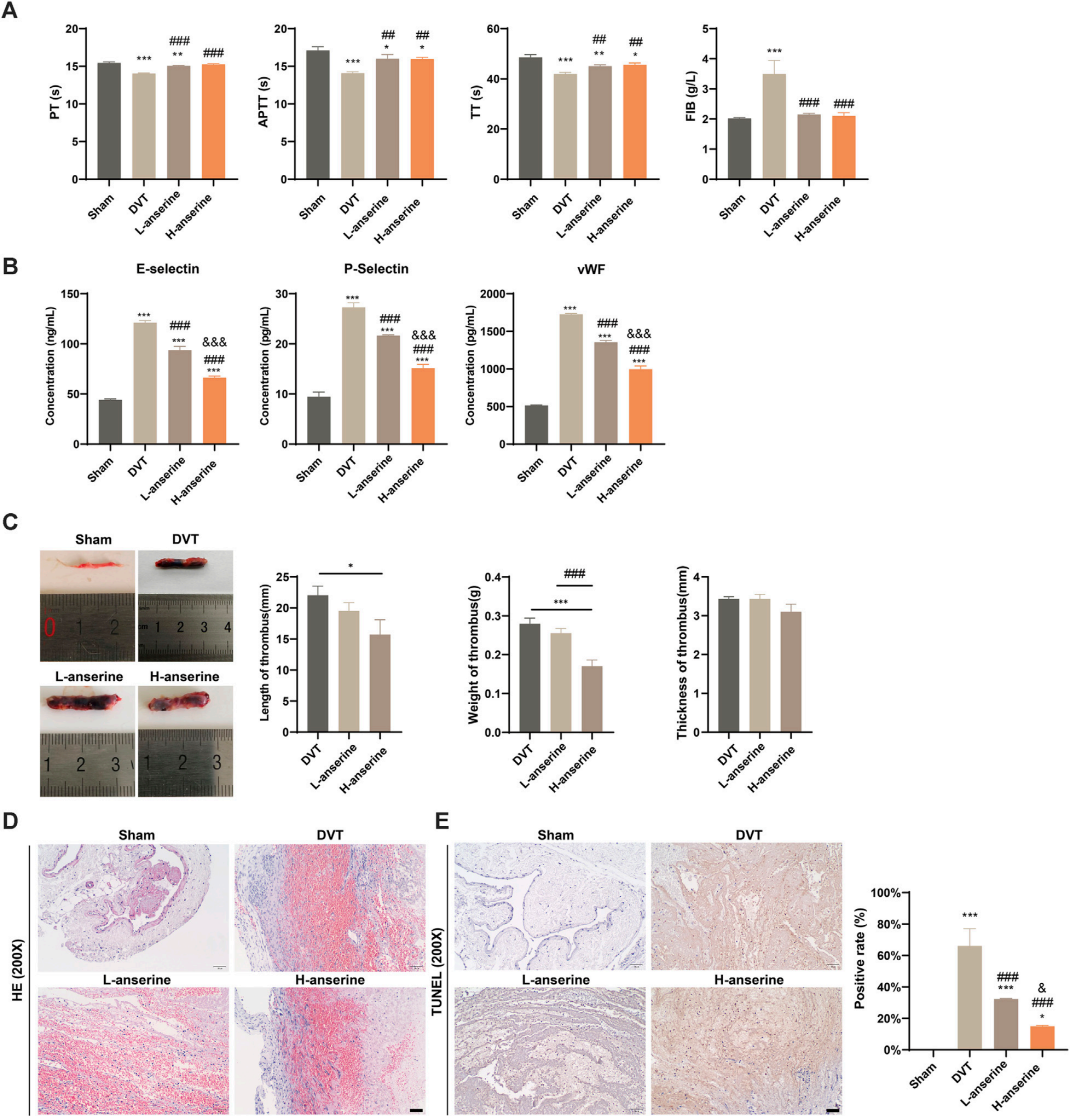
Vascular damage and thrombosis of inferior vena cava tissues in DVT rats are inhibited after 1 week of continuous gavage with anserine. **(A)** Four items of the coagulation tests (PT, TT, APTT, and FIB), **(B)** ELISA detection of serum P-selectin, E-selectin and vWF levels in each group, **(C–E)** In the tissues of inferior vena cava of each group, **(C)** thrombus length, weight and thickness, **(D)** pathological morphology, and **(E)** apoptosis are detected. Scale bar = 50 μm. Mean ± SD are shown (*n* = 3 in each group). *p < 0.05, **p < 0.01, ***p < 0.001 compared to the Sham group; ##*p* < 0.01, ###*p* < 0.001 compared to the DVT group; &&&*p* < 0.001 compared to the L-anserine group.

The effects of anserine on endothelial cell damage, adhesion molecule expression, and inflammation-related molecule expression were explored at the cellular level. To further investigate the effects of anserine on alleviating DVT *in vivo*, we constructed a rat model of DVT and administered different doses of anserine (3 and 15 mg/mL) to rats for 1 week, followed by four coagulation tests (Figure 3A). Both low- and high-dose anserine reduced serum P-selectin, E-selectin, and vWF levels in DVT rats (Figure 3B). *In vivo* experiments in DVT rats also showed that low and high doses of anserine inhibited thrombosis in the inferior vena cava tissue (Figure 3C). HE staining revealed the inhibitory effect of anserine on thrombosis in the tissues of inferior vena cava. Additionally, anserine treatment resulted in a reduction in the infiltration of inflammatory cells (Figure 3D). Furthermore, the anserine-treated groups exhibited a decrease in the number of apoptotic cells in the tissue (Figure 3E).

**FIGURE 4:**
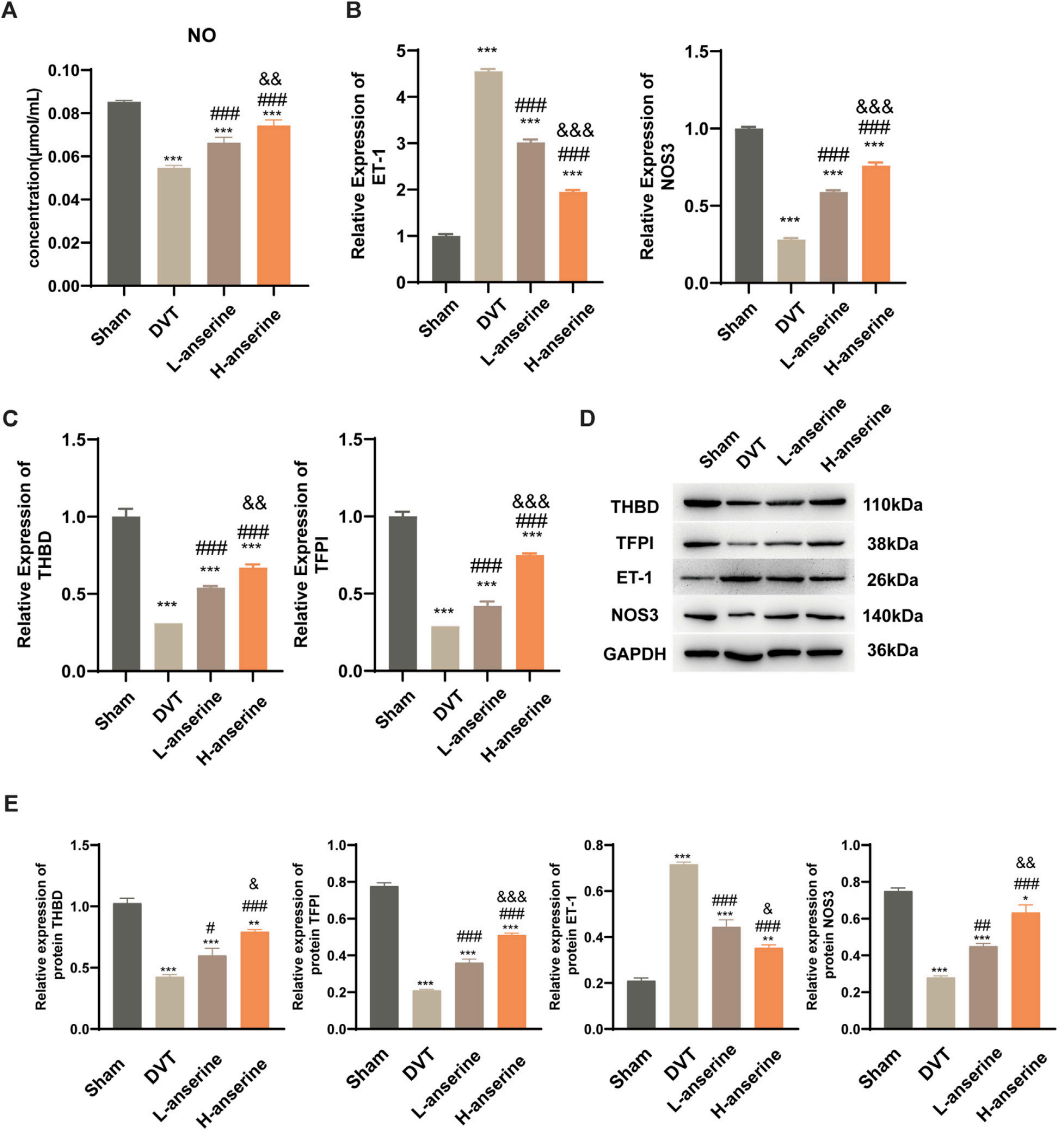
Continuous gavage with anserine after 1 week promotes vasodilation in DVT rats. **(A)** NO content determination in each group, **(B)** mRNA detection for vasoconstriction and relaxation-related molecules, **(C)** thrombosis-related molecules, and **(D, E)** expression of THBD, TFPI, ET-1, and NOS3 proteins using WB. Mean ± SD are shown (*n* = 3 in each group). *p < 0.05, **p < 0.01, ***p < 0.001 compared to the Sham group; #*p* < 0.05, ##*p* < 0.01, ###*p* < 0.001 compared to the DVT group; &*p* < 0.05, &&*p* < 0.01, &&&*p* < 0.001 compared to the L-anserine group.

After 1 week of continuous intragastric administration of anserine, NO release increased in DVT rats (Figure 4A), and ET-1 and NOS3 gene expression decreased and increased, respectively (Figure 4B). In addition, there was a significant increase in the expression of THBD and TFPI genes and proteins. On the other hand, the protein expression of ET-1 decreased, while the expression of NOS3 increased (Figures 4C–E).

### 3.4 Anserine relieves HUVEC damage by regulating MYB

**FIGURE 5:**
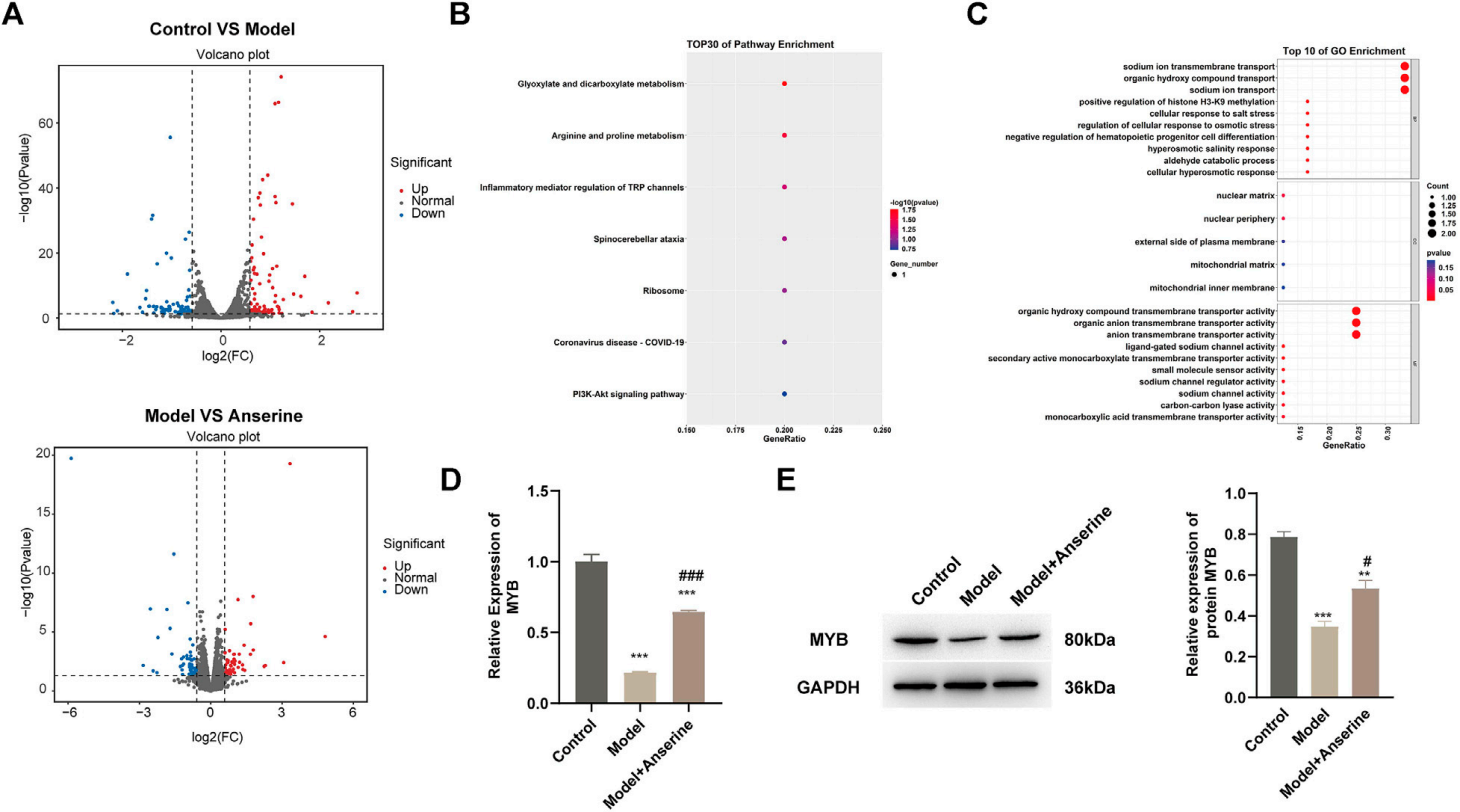
Transcriptome sequencing identifies MYB as a possible target gene for anserine in response to HUVEC injury. HUVECs were exposed to TNF-α (10 μg/L) for 24 h in the model group and the anserine treatment group. After the addition of anserine (500 μg/mL) to the culture medium, **(A)** volcano map of DEG analysis between the two groups, **(B, C)** overlapped genes that were differentially expressed in the model group and recurrent expression after treatment with anserine, **(B)** GO enrichment analysis, **(C)** KEGG pathway enrichment bubble charts, **(D)** qPCR detection of MYB gene expression **(E)** WB detection of MYB protein expression. Mean ± SD are shown (*n* = 3 in each group). ***p < 0.001 compared with the control group; #*p* < 0.05, ###*p* < 0.001 compared with the model group.

The results of both *in vivo* and *in vitro* experiments suggest that anserine mitigates HUVEC damage and DVT in rats. Next, we collected 500 μg/mL anserine-treated cells for transcriptome sequencing to explore the molecular mechanism of anserine in response to endothelial cell damage. Differentially expressed gene analyses after the treatment of HUVECs with TNF-α and anserine are shown in Figure 5A. We performed GO and KEGG analyses on genes differentially expressed in the model group and those in response to anserine treatment, and found that these genes mainly involved glyoxylate and dicarboxylate metabolism, arginine and proline metabolism, and the PI3K-Akt signaling pathway (Figure 5B). At the biological process level, genes were mainly related to the promotion of histone H3-K9 methylation and inhibition of hematopoietic progenitor cell differentiation (Figure 5C). Among these genes, MYB is also a transcriptional regulator of THBD and TFPI. Building upon previous research, it has been found that MYB plays a crucial role in regulating angiogenesis and platelet production (Carpinelli et al., 2004; Zhang et al., 2021). Therefore, MYB might be a key gene of anserine in response to HUVEC damage and DVT. In HUVECs, MYB gene and protein expression was significantly downregulated but significantly upregulated in the model group after anserine treatment (Figures 5D,E).

**FIGURE 6:**
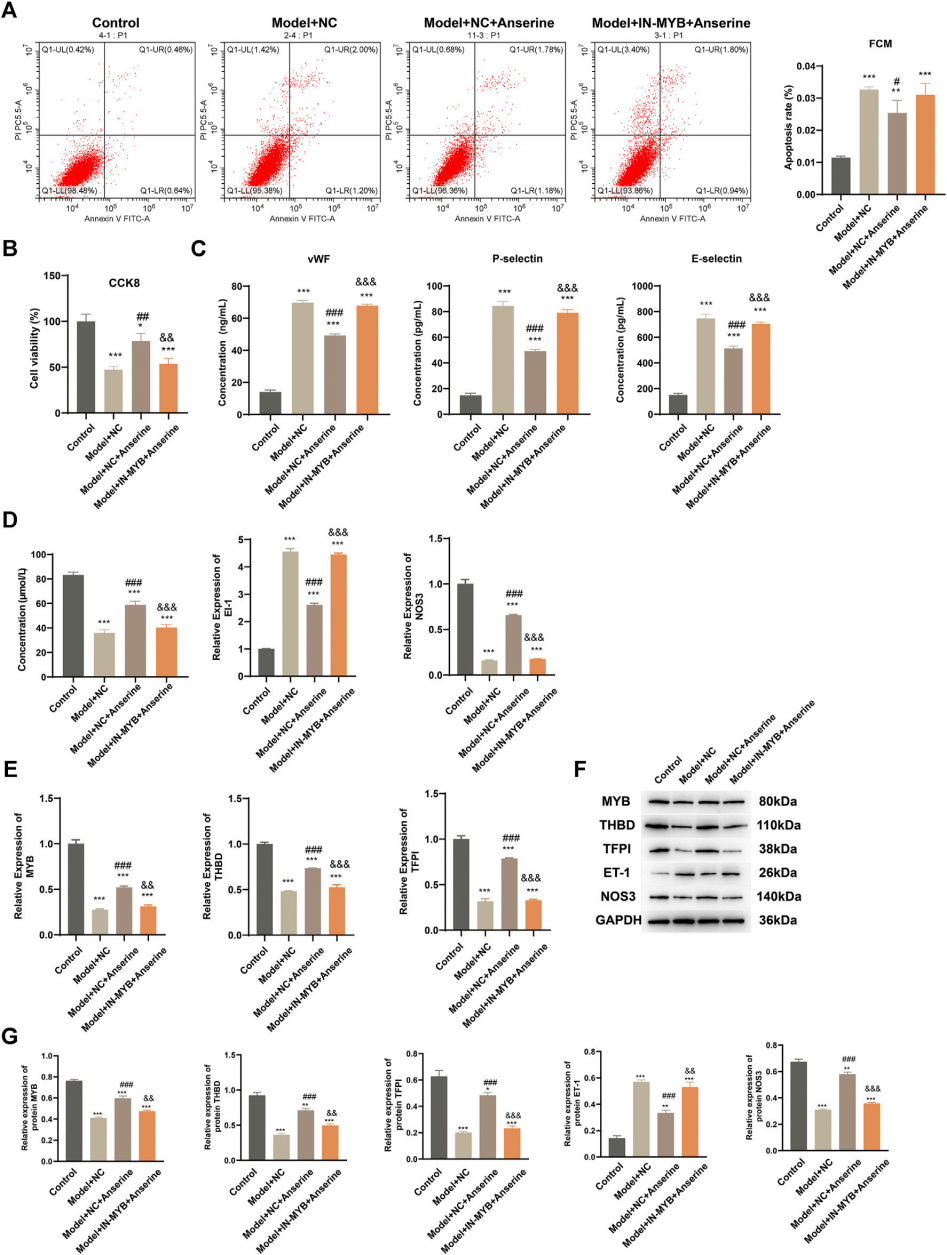
Interfering with MYB gene expression inhibits the effect of anserine on mitigating HUVEC injury. Relevant tests were performed in each group 72 h after MYB interference/empty-load lentivirus infection according to the grouping. **(A)** Apoptosis, **(B)** CCK8 detection of cell proliferation, **(C)** vWF, P-selectin, E-selectin assay, **(D)** detection of vasodilation and contraction-related molecules, **(E)** MYB, THBD, and TFPI gene expression levels, **(F, G)** MYB, THBD, TFPI, ET-1, and NOS3 protein expression. Mean ± SD are shown (*n* = 3 in each group). *p < 0.05, **p < 0.01, ***p < 0.001 compared with the Control group; #*p* < 0.05, ##*p* < 0.01, ###*p* < 0.001 compared with the Model + NC group; &&*p* < 0.01, &&&*p* < 0.001 compared with the Model + NC + Anserine group.

The MYB interference/empty-load lentivirus infection group was further set up according to the grouping to explore the regulatory effect of MYB on HUVECs injury. In comparison to the Model + NC + Anserine group, the apoptosis of the Model + IN-MYB + Anserine group was significantly increased (Figure 6A), whereas cell proliferation was significantly reduced (Figure 6B). Furthermore, in the IN-MYB group, the levels of cell vWF, P-selectin, and E-selectin were significantly increased (Figure 6C). In comparison to Model + NC + Anserine, NO release was significantly downregulated in the Model + IN-MYB + Anserine group, and ET-1 and NOS3 gene expression were upregulated and downregulated in the Model + IN-MYB + Anserine group, respectively (Figure 6D). In addition, IN-MYB infection significantly reduced THBD and TFPI gene and protein expression, and MYB and NOS3 protein expression, but ET-1 protein expression was significantly increased (Figures 6E–G).

### 3.5 CARNMT1 alleviates DVT rats by controlling the metabolic level of anserine

**FIGURE 7:**
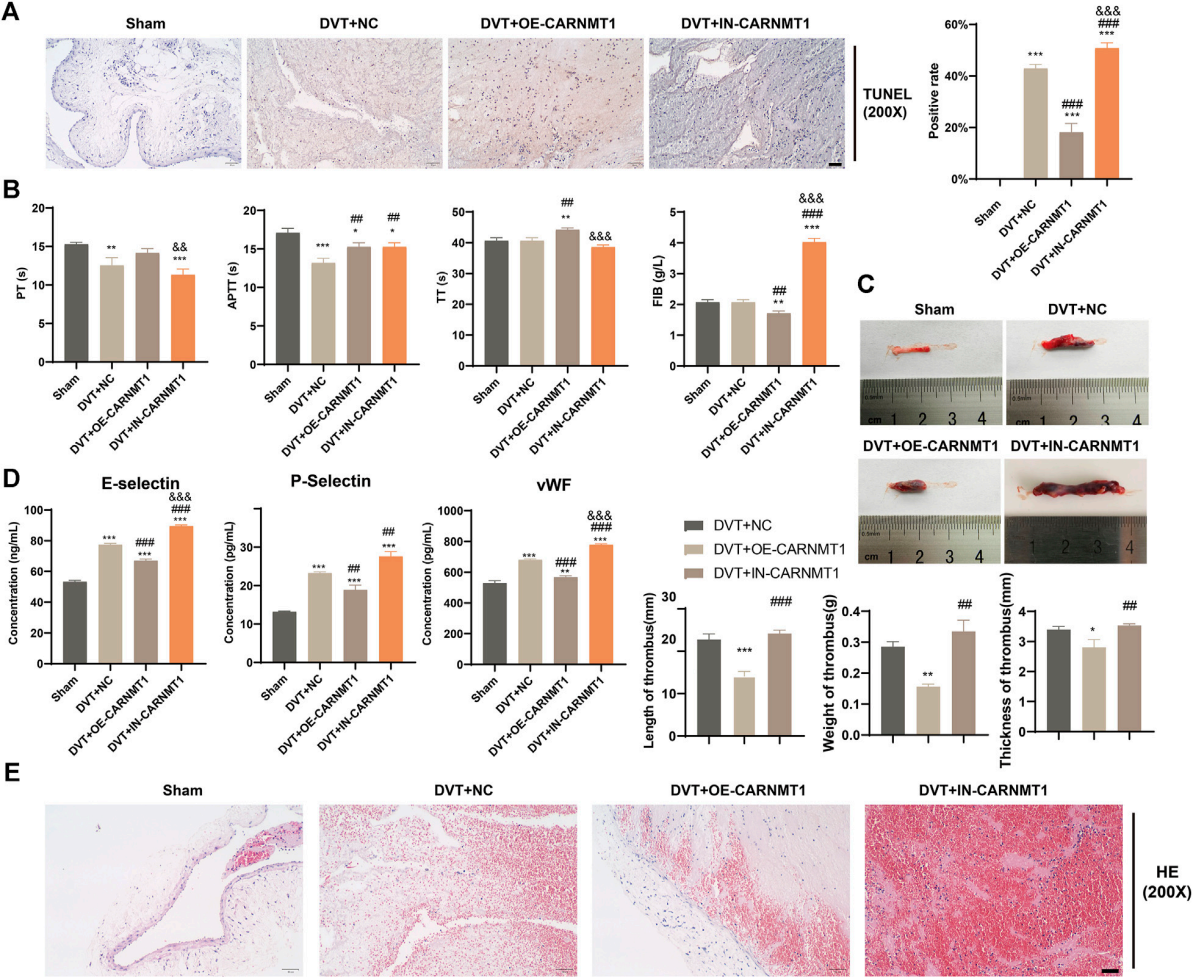
Effects of CARNMT1 on the injury of inferior vena cava tissue cells and thrombosis in DVT rats. **(A)** Tunel detects histiocyte apoptosis, Scale bar = 50 μm, **(B)**. Four items of blood coagulation tests in each group: PT, TT, APTT, and FIB, **(C)** Thrombosis morphology in each group of rats, **(D)** ELISA detects the contents of vWF, P-selectin, and E-selectin, **(E)** Detection of thrombus length, weight and thickness in each group of rats, Scale bar = 50 μm. Mean ± SD are shown (*n* = 3 in each group). *p < 0.05, **p < 0.01, ***p < 0.001 compared to the Sham group; ##*p* < 0.01, ###*p* < 0.001 compared to the DVT + NC group; &*p* < 0.05, &&*p* < 0.01, &&&*p* < 0.001 compared to the DVT + OE-CARNMT1 group.

**FIGURE 8:**
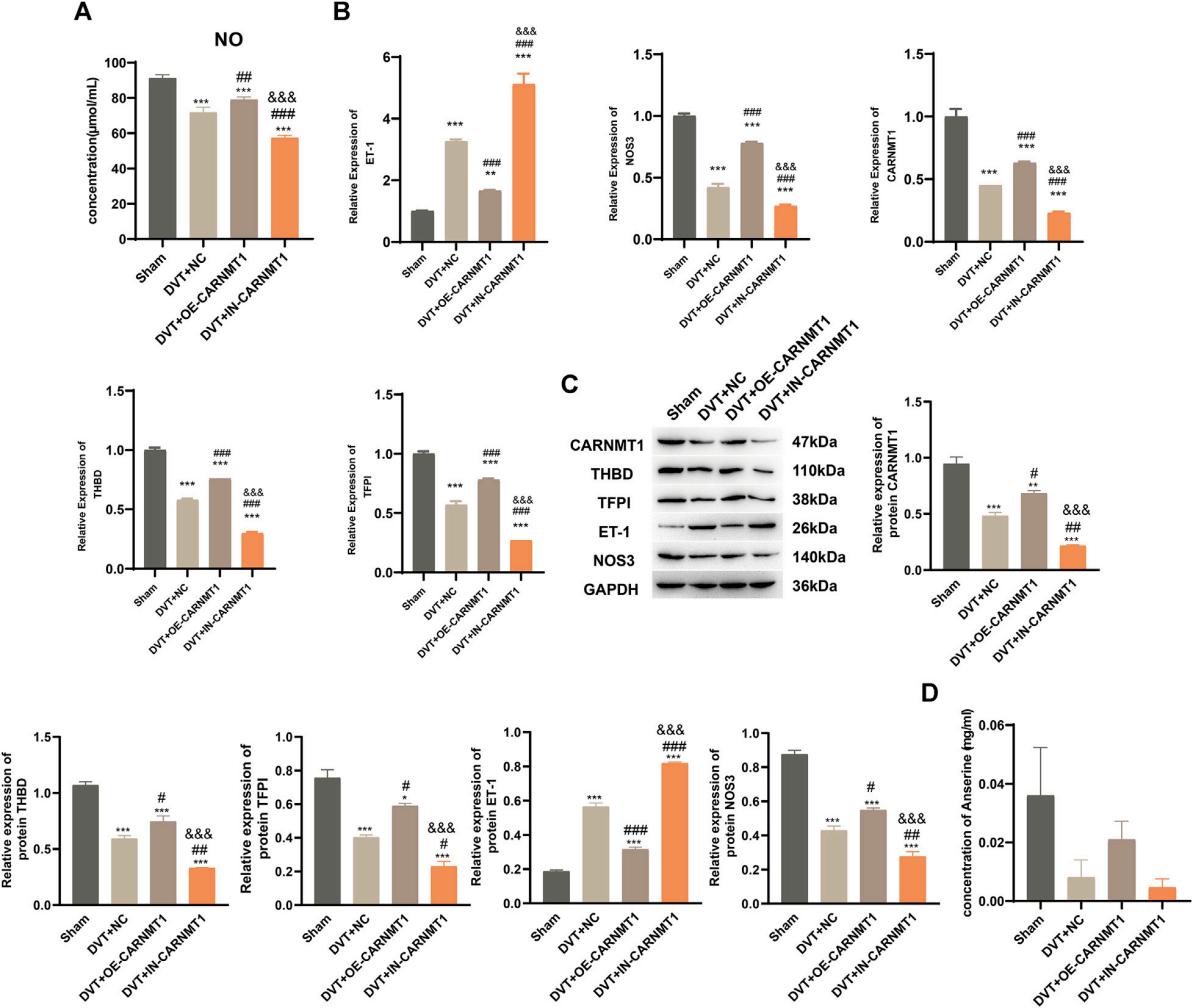
CARNMT1 promotes anserine metabolism in DVT rats. CARNMT1 interfering/overexpressing adeno-associated virus tail vein injection was performed according to grouping, and tissues were collected 1 week later for relevant testing. **(A)** Detection of molecules related to vasodilation and contraction. **(B)** CARNMT1, THBD, and TFPI gene expression levels. **(C)** HPLC detects the metabolic content of anserine in rats. **(D, E)** CARNMT1, THBD, TFPI, ET-1, and NOS3 protein expression. Mean ± SD are shown (*n* = 3 in each group). *p < 0.05, **p < 0.01, ***p < 0.001 compared with the Sham group; #*p* < 0.05, ##*p* < 0.01, ###*p* < 0.001 compared with the DVT + NC group; &&&*p* < 0.001 compared with the DVT + OE-CARNMT1 group.

According to the KEGG metabolic pathway database (http://www.kegg.jp), the synthetic rate-limiting enzyme CARNMT1 of the metabolite anserine was used to overexpress CARNMT1 and interfere with adeno-associated viruses. The rats in each experimental group were administered tail vein injections. After 1 week of continuous intragastric administration of anserine, OE-CARNMT1 inferior vena cava tissue cell apoptosis was reduced (Figure 7A). The effect of CARNMT1 on coagulation in rats is shown in Figure 7B. OE-CARNMT1 tail vein injection inhibited thrombosis and tissue inflammatory infiltration in rats (Figures 7C,E). And vWF, P-selectin, and E-selectin levels were significantly reduced (Figure 7D). In addition, in the DVT + OE-CARNMT1 group, NO release increased, ET-1 and NOS3 gene expression decreased and increased, respectively (Figures 8A,B). CARNMT1, THBD, and TFPI gene and protein expression significantly increased, while ET-1 and NOS3 protein expression decreased and increased, respectively (Figures 8B,C). The anserine metabolite content was significantly higher in the DVT + OE-CARNMT1 group (Figure 8D).

## 4 Discussion

DVT refers to abnormal formation of blood clots within the deep vein, leading to venous return obstruction. Common thrombotic diseases include pulmonary thromboembolism, ischemic stroke, venous thrombosis, myocardial infarction, and coronary heart disease, which have high incidences and can be life-threatening (Akers et al., 2019). Patients may experience clinical symptoms such as pain in the extremities, swelling, and excessive epidermal temperature. Once a deep vein thrombus is formed, the embolus is prone to fall off, which may enter the pulmonary artery with blood circulation, causing pulmonary artery embolism and even death of the patient due to pulmonary embolism (McCullough et al., 2018). In addition, if DVT is not treated in a timely and effective manner, it will develop into post-thrombotic syndrome and even lead to disability, which seriously affects the prognosis of patients (Galanaud et al., 2018). As a major emerging research field in systems biology, metabolomics can comprehensively reflect the metabolic state of the body under pathological conditions and deeply understand the mechanism of thrombosis.

In this study, we conducted an analysis of the KEGG pathway of differential metabolites and differential metabolite enrichment between the DVT group and the sham group. The experimental results revealed significant alterations in the serum content of many metabolites in the DVT group. Among these, anserine was identified as a downregulated differential metabolite in DVT when analyzed in the cationic mode. Gu et al. identified reduced levels of metabolites in DVT rats using ultra-performance liquid chromatography based on metabolomics (Gu et al., 2022). Anserine is a multifunctional and highly stable dipeptide analogue of carnosine. Research by Peters et al. suggests that short-term treatment with anserine can improve vascular permeability in diabetic mice (Peters et al., 2018). Anserine has been reported to play a significant physiological role in antioxidant and anti-inflammatory responses (Wu, 2020), and its downregulation in the DVT model group may prevent DVT rats from playing a regulatory role in the inflammatory response after venous thrombosis (Katakura et al., 2017). Enrichment analysis based on the KEGG metabolic pathway was used to determine the alterations in the metabolic pathways associated with the differential metabolites. The observed differences in the metabolic pathways between the DVT model rats and the sham-operated group were mainly concentrated in histidine metabolism, which contains the differential metabolite goosomyopeptide.

In this study, the mitigation effect of anserine on DVT was studied in many aspects, including the mitigation effect on endothelial cell injury and the modulation effect on blood coagulation function and vascular injury in DVT rats. In the HUVECs endothelial injury model, it was found that anserine significantly inhibited apoptosis, enhanced cell proliferation, and played a positive role in vasodilatation by inhibiting and enhancing ET-1 and NOS3 gene and protein expression, respectively, while significantly reducing P-selectin and E-selectin molecular levels, inhibiting inflammatory response, and significantly inhibiting vWF release levels, THBD, and TFPI gene and protein expression, thereby inhibiting platelet aggregation and activation and reducing thrombosis (Yang et al., 2017; Manderstedt et al., 2022). In addition, the positive effects of anserine on endothelial damage, blood agglutination, and the inflammatory response in rats have also been demonstrated in rats with DVT. Vascular endothelial cells are distributed between the subcutaneous tissues of the vascular wall and have various biological functions. Under normal physiological conditions, vascular endothelial cells can inhibit coagulation factors and platelet activation, secrete fibrinolytic molecules, and prevent thrombosis. Once vascular endothelial cells are damaged by external factors, they can promote thrombosis, resulting in impaired biological functions (Lau et al., 2022). There has been a growing focus on the regulatory role of vascular endothelial cells in DVT formation in recent years (Bochenek and Schäfer, 2019). Some scholars have proposed that protecting the function of endothelial cells and promoting their repair should be new ideas for the clinical treatment of DVT (Rajendran et al., 2013). Research has shown that increasing MYB expression can promote tube formation of brain microvascular endothelial cells, indicating a crucial role of MYB in regulating angiogenesis (Zhang et al., 2021). Additionally, knocking down MYB enhances platelet production, and platelets are critical factors in blood coagulation, suggesting that MYB may play a role in DVT (Carpinelli et al., 2004).

To gain a deeper understanding of the molecular mechanism underlying the effects of anserine on the biological function of HUVECs, we revealed the molecular mechanism of anserine in response to endothelial cell injury through transcriptome sequencing and gene expression analysis and found that MYB may be a key gene of anserine in response to HUVECs injury and DVT, which was confirmed through MYB interference experiments. Transcriptomic sequencing analysis using the KEGG pathway revealed that the PI3K-Akt signaling pathway is enriched with the MYB gene. In endothelial cells, activation of Akt promotes cell survival, suggesting that the PI3K/Akt pathway may play a crucial role in regulating various inflammatory diseases (Tao et al., 2019). Inhibiting the PI3K/Akt/NF-кB pathway leads to a reduction in the expression of IL-1β, IL-6, and TNF-α, thereby improving the inflammatory environment and survival of HUVECs (Baratchi et al., 2017). In addition, the effect of CARNMT1 on the regulation of anserine metabolism in response to DVT was studied, and its effect on DVT rats was confirmed by experiments. The protein encoded by CARNMT1 is a methyltransferase that converts carnosine to anserine (Cao et al., 2018). The findings demonstrate that overexpression of CARNMT1 in DVT rats can increase the anserine content, prolong PT, TT, and aPTT, inhibit FIB, and promote the anticoagulant function of rats. The results also showed that overexpression of CARNMT1 inhibited vWF, P-selectin, and E-selectin content; reduced THBD and TFPI gene and protein expression; and inhibited thrombosis and inflammatory infiltration in rats. Based on these findings, it can be inferred that CARNMT1 shows potential as a therapeutic target for DVT owing to its modulation of anserine content and inhibition of blood clotting, inflammatory response, and thrombosis in DVT rats.

These results suggest that anserine could potentially serve as a therapeutic agent for DVT management. Its ability to decrease adhesion molecule expression and inflammation, highlights its multifaceted benefits in mitigating DVT progression. Translating experimental interventions such as anserine supplementation and CARNMT1 modulation into clinical practice poses several challenges but also holds significant promise. Conducting rigorous clinical trials to evaluate the safety, efficacy, and optimal dosage of anserine supplementation in DVT patients is essential. This study also has some limitations. It should be noted that although the current findings systematically elucidate the multifaceted mitigating effects of anserine on DVT, the results are mutually corroborated by cellular and animal experiments. In addition, the molecular mechanism of anserine in DVT has been revealed, providing preliminary evidence for the association of the MYB gene with DVT. It has been determined that CARNMT1, as a regulatory enzyme of anserine metabolites, has a mitigating effect on DVT. These findings offer a crucial theoretical foundation for future development of related drugs and treatments in the future. However, further research is warranted to explore and verify the exact mechanisms through which MYB and CARNMT1 exert their effects in the context of DVT.

## 5 Conclusion

The findings of this study demonstrated that anserine has a crucial regulatory role in HUVECs injury and thrombosis in DVT rats. It functions by regulating the expression of adhesion molecules, inhibiting thrombosis, reducing apoptosis, and promoting NO release. Further transcriptome sequencing and genetic intervention results suggested that anserine may modulate the activity of the PI3K-Akt signaling pathway and coagulation function through MYB. Additionally, CARNMT1 was found to play a role in the modulation of anserine metabolism.

## Data Availability

The original contributions presented in the study are publicly available. This data can be found in the OMIX, China National Center for Bioinformation/Beijing Institute of Genomics, Chinese Academy of Sciences (https://ngdc.cncb.ac.cn/omix: accession no.OMIX006390).
