# Supplementary material for: Exploration on the effect of anserine on the alleviation of DVT and its molecular mechanism
Source: Front Pharmacol. 2024 May 23;15:1402758. doi: 10.3389/fphar.2024.1402758 (PMC11154784; doi:10.3389/fphar.2024.1402758)
Supplement: Supplementary file 1 [file Table1.docx]

Supplementary Material

# Supplementary Tables

Supplementary Table 1 Fluorescent quantitative PCR primer sequences

| Primer names | Sequences |
| --- | --- |
| h-THBD-F | GGGCTTGCTCATAGGCATCT |
| h-THBD-R | GCAGCACTACCTCCTTGGAA |
| h-TFPI-F | GGTCCGAATGGTTTCCAGGT |
| h-TFPI-R | ACCTTGGTTGATTGCGGAGT |
| h-ET-1-F | CAAACCAGGTCGGAGACCAT |
| h-ET-1-R | GCTCGGTTGTGGGTCACATA |
| h-NOS3-F | GAAGCGAGTGAAGGCGACAA |
| h-NOS3-R | AACTCTTGTGCTGTTCCGGC |
| h-MYB-F | GCAGGTGCTACCAACACAGA |
| h-MYB-R | CGAGGCGCTTTCTTCAGGTA |
| h-GAPDH-F | GTCAAGGCTGAGAACGGGAA |
| h-GAPDH-R | AAATGAGCCCCAGCCTTCTC |
| r-THBD-F | CCTTTGTCTTTCCGGGCTCT |
| r-THBD-R | TCAAGTCCTCCCTACCCTCG |
| r-TFPI-F | GGCCCTTCATGGTGTCTTGA |
| r-TFPI-R | TCCCCCACATCCAGTGTAGT |
| r-ET-1-F | CCAGCGTCCTTGTTCCAAAC |
| r-ET-1-R | TCACTTGCTACCAGCGGATG |
| r-NOS3-F | TGCGTCGCTTCATTAGGTTG |
| r-NOS3-R | CAAAGCATACGAAGAGGGCAG |
| r-GAPDH-F | AGAGACAGCCGCATCTTCTT |
| r-GAPDH-R | GGTAACCAGGCGTCCGATAC |

Supplementary Table 2 Primary antibody used in Western blot (WB)

| Primary antibody | Dilution ratio | Manufacturer | Item No. |
| --- | --- | --- | --- |
| THBD | 1:1000 | China, abclonal | A4155 |
| TFPI | 1:1000 | China, abclonal | A8704 |
| ET-1 | 1:1000 | China, abclonal | A0686 |
| NOS3 | 1:1000 | China, abclonal | A1548 |
| GAPDH | 1:1000 | China, abclonal | A19056 |

Supplementary Table 3 Interfering target site sequences

| Target site | Sequence |
| --- | --- |
| MYB-oligo | CCAGAATCTCTCAAGCGTGAA |
| Top Strand | 5'-CGCCAGAATCTCTCAAGCGTGAACTCGAGTTCACGCTTGAGAGATTCTGGTTTTTTG-3' |
| Bottom Strand | 5'-AATTCAAAAAACCAGAATCTCTCAAGCGTGAACTCGAGTTCACGCTTGAGAGATTCTGGCG-3' |

Supplementary Table 4 CARNMT1 interference target site sequence

| Target site | Sequence |
| --- | --- |
| CARNMT1-oligo | GAATCTGTATTGTCAACATAT |
| Top Strand | 5'-TGCAgGGAATCTGTATTGTCAACATATCTCGAGATATGTTGACAATACAGATTCTTTTTTg-3' |
| Bottom Strand | 5'-cAAAAAAGAATCTGTATTGTCAACATATCTCGAGATATGTTGACAATACAGATTCCctgca-3' |

Supplementary Table 5. Differential metabolites that were significant changed in Sham/Model of serum

| SuperClass | Class | Name | VIP | P value | Up/Down |
| --- | --- | --- | --- | --- | --- |
| Benzenoids | Benzene and substituted derivatives | 2-(3,4-dimethoxyphenyl)ethanamine | 1.229540665 | 0.0000 | down |
|  |  | 11-trans Leukotriene C4 | 1.123719112 | 0.0001 | up |
|  |  | o-Toluic Acid | 1.075628774 | 0.0001 | up |
|  |  | Hippuric acid | 1.502839099 | 0.0000 | down |
|  |  | N-Benzylformamide | 1.160901288 | 0.0001 | up |
|  |  | Hordenine | 1.245391786 | 0.0002 | up |
|  | Phenols | Hydroquinone | 1.424882543 | 0.0000 | down |
|  |  | Isoproterenol | 1.141435203 | 0.0000 | down |
|  |  | 2-Methoxyresorcinol | 1.385204171 | 0.0000 | down |
| Lipids and lipid-like molecules | Endocannabinoids | 2-Arachidonyl Glycerol ether | 1.513353456 | 0.0000 | up |
|  | Fatty Acyls | Citraconic acid | 1.401307954 | 0.0000 | down |
|  |  | Stearic acid | 1.376899144 | 0.0000 | up |
|  |  | Elaidic acid | 1.350003897 | 0.0000 | up |
|  |  | Prostaglandin D2 | 1.287009298 | 0.0000 | down |
|  |  | 2-Hydroxyvaleric acid | 1.351035367 | 0.0000 | up |
|  |  | Docosahexaenoic acid | 1.283121821 | 0.0000 | up |
|  |  | Myristic acid | 1.274547547 | 0.0000 | up |
|  |  | Dihydroroseoside | 1.282693393 | 0.0000 | down |
|  |  | 2-Hydroxymyristic acid | 1.251326928 | 0.0000 | up |
|  |  | 5-OxoETE | 1.245426432 | 0.0000 | up |
|  |  | Palmitic acid | 1.293245789 | 0.0000 | up |
|  |  | 13Z,16Z-Docosadienoic Acid | 1.215242211 | 0.0000 | up |
|  |  | 12(S)-HETE | 1.199724435 | 0.0000 | up |
|  |  | 2-Hydroxycaproic acid | 1.290052921 | 0.0000 | up |
|  |  | Mesaconic acid | 1.198157554 | 0.0000 | down |
|  |  | Suberic acid | 1.180665332 | 0.0000 | up |
|  |  | 16-Hydroxyhexadecanoic acid | 1.216252854 | 0.0000 | up |
|  |  | 2-Methylbutyric acid | 1.141453247 | 0.0000 | up |
|  |  | Lignoceric Acid | 1.157574563 | 0.0000 | up |
|  |  | 3-Methyl-2-oxobutanoic acid | 1.114167613 | 0.0000 | up |
|  |  | Prostaglandin B2 | 1.076290293 | 0.0001 | down |
|  |  | Sebacic acid | 1.075716262 | 0.0001 | down |
|  |  | Adrenic acid | 1.086419798 | 0.0001 | up |
|  |  | Docosapentaenoic acid | 1.100459358 | 0.0001 | up |
|  |  | Lauric Acid | 1.034183206 | 0.0001 | up |
|  |  | Dodecanedioic acid | 1.055476855 | 0.0001 | down |
|  |  | Nonadecanoic acid | 1.005981683 | 0.0002 | up |
|  |  | Jasmonic acid | 1.015818996 | 0.0004 | down |
|  |  | Prostaglandin A1 | 1.044328605 | 0.0005 | up |
|  |  | Azelaic acid | 1.024487424 | 0.0015 | down |
|  |  | 10-Nitrolinoleate | 1.521407327 | 0.0000 | down |
|  |  | O-Arachidonoyl ethanolamine | 1.382200632 | 0.0000 | up |
|  |  | Decanoylcarnitine | 1.366703284 | 0.0000 | up |
|  |  | Hexanoylcarnitine | 1.380980652 | 0.0000 | up |
|  |  | Acetyl-L-carnitine | 1.384434415 | 0.0000 | up |
|  |  | Palmitoylcarnitine | 1.379807798 | 0.0000 | up |
|  |  | 3-Methyladipic acid | 1.313632078 | 0.0000 | down |
|  |  | 13-OxoODE | 1.267310623 | 0.0000 | down |
|  |  | 8,15-Dihete | 1.187624512 | 0.0002 | down |
|  |  | Carbaprostacyclin | 1.222461609 | 0.0005 | down |
|  | Prenol lipids | Diosgenin | 1.041368871 | 0.0066 | down |
|  | Steroids and steroid derivatives | Lithocholic Acid | 1.334181323 | 0.0000 | down |
|  |  | Androsterone | 1.171411779 | 0.0000 | up |
|  |  | Estriol | 1.239075686 | 0.0000 | down |
|  |  | Methandrostenolone | 1.273371764 | 0.0000 | up |
|  |  | Deoxycholic acid | 1.145423043 | 0.0002 | down |
|  |  | Taurocholic acid | 1.197374014 | 0.0002 | up |
|  |  | Glycoursodeoxycholic acid | 1.461820827 | 0.0000 | down |
|  |  | Tetrahydrocorticosterone | 1.376475607 | 0.0000 | up |
|  |  | Taurodeoxycholic Acid | 1.382167752 | 0.0000 | down |
|  |  | Pregnenolone | 1.334319676 | 0.0001 | down |
|  |  | 17alpha-Hydroxyprogesterone | 1.288305857 | 0.0001 | down |
|  |  | Progesterone | 1.293204681 | 0.0001 | down |
| Nucleosides, nucleotides, and analogues | 5'-deoxyribonucleosides | 5'-Deoxy-5'-(Methylthio)Adenosine | 1.201638844 | 0.0000 | up |
|  | Purine nucleotides | Adenosine diphosphate ribose | 1.317379654 | 0.0000 | up |
|  |  | dAMP | 1.582070641 | 0.0000 | down |
|  | Pyrimidine nucleosides | Deoxycytidine | 1.2308555 | 0.0000 | up |
|  |  | 5-Methyl-2'-deoxycytidine | 1.358546197 | 0.0000 | down |
|  |  | 5-Methyluridine | 1.07605057 | 0.0008 | down |
|  | Pyrimidine nucleotides | UDP-galactose | 1.402621966 | 0.0000 | up |
| Organic acids and derivatives | Carboximidic acids and derivatives | Palmitoyl ethanolamide | 1.442719404 | 0.0000 | up |
|  | Carboxylic acids and derivatives | Citric acid | 1.47176771 | 0.0000 | down |
|  |  | 4-Oxoproline | 1.39143004 | 0.0000 | up |
|  |  | L-serine | 1.387745114 | 0.0000 | up |
|  |  | Arginine | 1.383172855 | 0.0000 | up |
|  |  | L-Glutamate | 1.352203784 | 0.0000 | up |
|  |  | L-Asparagine | 1.315441044 | 0.0000 | up |
|  |  | 4-Aminobutyric acid | 1.342669178 | 0.0000 | up |
|  |  | gamma-Glutamylmethionine | 1.262782915 | 0.0000 | up |
|  |  | Cystine | 1.276358828 | 0.0000 | up |
|  |  | D-Phenylalanine | 1.238518547 | 0.0000 | up |
|  |  | Acetylcysteine | 1.232816848 | 0.0000 | down |
|  |  | Trichloroacetic acid | 1.112747561 | 0.0000 | up |
|  |  | Threonine | 1.197488287 | 0.0000 | up |
|  |  | L-cysteine | 1.203266282 | 0.0000 | up |
|  |  | Phenylacetylglycine | 1.059279969 | 0.0001 | down |
|  |  | Tiglylglycine | 1.051835003 | 0.0001 | down |
|  |  | cis-Aconitic acid | 1.077674594 | 0.0002 | up |
|  |  | α-Aspartylphenylalanine | 1.000083133 | 0.0002 | down |
|  |  | N-Acetyl-D-tryptophan | 1.015553204 | 0.0002 | up |
|  |  | 3-Hydroxy-L-proline | 1.105864416 | 0.0004 | down |
|  |  | Methionine | 1.670230865 | 0.0000 | up |
|  |  | Tazobactam sodium | 1.474907439 | 0.0000 | up |
|  |  | Levothyroxine | 1.454144495 | 0.0000 | down |
|  |  | L-Glutamic acid | 1.626177573 | 0.0000 | up |
|  |  | 3,3',5-Triiodo-L-thyronine | 1.482276765 | 0.0000 | up |
|  |  | D-Ala-D-Ala | 1.398424598 | 0.0000 | down |
|  |  | 4-Guanidinobutanoic acid | 1.382191391 | 0.0000 | down |
|  |  | Creatine | 1.400928216 | 0.0000 | up |
|  |  | L-Hydroxyproline | 1.311838885 | 0.0000 | down |
|  |  | Betaine | 1.287601542 | 0.0000 | down |
|  |  | N-Phenylacetylglutamine | 1.255599211 | 0.0000 | down |
|  |  | N-Acetylornithine | 1.24013667 | 0.0000 | down |
|  |  | N-Acetylvaline | 1.229040869 | 0.0000 | down |
|  |  | trans-4-Hydroxy-L-proline | 1.369899789 | 0.0001 | down |
|  |  | DL-Arginine | 1.211431765 | 0.0001 | up |
|  |  | 4-Acetamidobutyric Acid | 1.193696087 | 0.0001 | down |
|  |  | Proline-hydroxyproline | 1.213632837 | 0.0002 | up |
|  |  | Leu-Pro | 1.258307153 | 0.0004 | down |
|  |  | Gly-Phe | 1.044348447 | 0.0009 | down |
|  |  | Oxaceprol | 1.145201196 | 0.0044 | down |
|  | Hydroxy acids and derivatives | 3-Hydroxybutyric acid | 1.127272826 | 0.0000 | up |
|  |  | 10-Hydroxydecanoic acid | 1.170130834 | 0.0000 | up |
|  | Keto acids and derivatives | 4-Methyl-2-Oxopentanoic Acid | 1.173388038 | 0.0000 | up |
|  |  | 2-Oxobutyric acid | 1.053989251 | 0.0001 | up |
|  |  | 2-Oxoglutaric acid | 1.045462608 | 0.0003 | up |
|  |  | alpha-Ketoglutaric acid | 1.749874678 | 0.0000 | down |
|  | Organic phosphoric acids and derivatives | TPP | 1.232808371 | 0.0000 | up |
|  | Organic sulfuric acids and derivatives | 3-Indoxyl sulphate | 1.370566034 | 0.0000 | down |
|  | Peptidomimetics | Anserine | 1.372556646 | 0.0009 | down |
| Organic nitrogen compounds | Organonitrogen compounds | Histamine | 1.453234857 | 0.0000 | down |
|  |  | DL-Carnitine | 1.388349726 | 0.0000 | up |
|  |  | Spermidine | 1.155334864 | 0.0001 | up |
| Organic oxygen compounds | Organooxygen compounds | Pantothenic acid | 1.480541065 | 0.0000 | up |
|  |  | D-Threose | 1.307492201 | 0.0000 | up |
|  |  | D-Ribose-1-phosphate | 1.165453601 | 0.0000 | up |
|  |  | α-D-Mannose 1-phosphate | 1.214275304 | 0.0000 | up |
|  |  | 5'-Adenylic acid | 1.123919183 | 0.0000 | down |
|  |  | D-Glucosamine 6-phosphate | 1.133886037 | 0.0000 | down |
|  |  | Chlorogenic acid | 1.402805147 | 0.0000 | down |
|  |  | Linustatin | 1.398232117 | 0.0000 | down |
|  |  | N-Formylkynurenine | 1.202453283 | 0.0001 | up |
|  |  | Trehalose | 1.247494711 | 0.0006 | down |
|  |  | D-Raffinose | 1.114438505 | 0.0010 | down |
|  |  | D-(+)-Maltose | 1.184878783 | 0.0037 | down |
|  |  | Gluconolactone | 1.038453204 | 0.0057 | down |
| Organoheterocyclic compounds | Azoles | Methylimidazoleacetic acid | 1.420636627 | 0.0000 | down |
|  |  | Imidazoleacetic acid | 1.360212262 | 0.0000 | down |
|  | Diazines | Orotic Acid | 1.141361567 | 0.0000 | up |
|  |  | N-Oleoyl Glycine | 1.120451461 | 0.0000 | up |
|  |  | Dihydrothymine | 1.403735797 | 0.0000 | down |
|  |  | Tetramethylpyrazine | 1.000077484 | 0.0065 | up |
|  | Dihydrofurans | Ascorbic acid | 1.298240637 | 0.0000 | up |
|  | Dithiolanes | Lipoic acid | 1.212656836 | 0.0000 | up |
|  | Furans | 2,5-Furandicarboxylic acid | 1.713953741 | 0.0000 | down |
|  | Indoles and derivatives | 3-Methylindole | 1.291860013 | 0.0000 | up |
|  |  | Indole-3-lactic acid | 1.115900599 | 0.0000 | up |
|  |  | 5-Hydroxytryptophol | 1.641190881 | 0.0000 | down |
|  |  | 3-Indoleacrylic acid | 1.519023784 | 0.0000 | down |
|  |  | Methyl indole-3-acetate | 1.643719465 | 0.0000 | down |
|  |  | 5-Hydroxyindole-3-acetic acid | 1.361197975 | 0.0000 | down |
|  |  | 5-Hydroxyindole | 1.344754491 | 0.0000 | down |
|  |  | Indole-3-acetic acid | 1.153247634 | 0.0002 | down |
|  |  | Serotonin | 1.007508791 | 0.0029 | down |
|  | Isobenzofurans | Sedanolide | 1.154778277 | 0.0040 | down |
|  | Isoquinolines and derivatives | Isoquinoline | 1.640402586 | 0.0000 | down |
|  | Naphthofurans | Cafestol | 1.185402533 | 0.0002 | down |
|  | Pteridines and derivatives | Vitamin B2 | 1.352918041 | 0.0000 | up |
|  |  | Riboflavin | 1.310189051 | 0.0001 | up |
|  | Pyridines and derivatives | Pyridoxine | 1.304770021 | 0.0000 | down |
|  |  | Nicotinamide | 1.742209638 | 0.0000 | down |
|  |  | Pyridoxamine 5-phosphate | 1.388578593 | 0.0000 | up |
|  |  | 6-Methylnicotinamide | 1.180458033 | 0.0061 | down |
|  | Pyrroles | Pyrrole-2-carboxylic acid | 1.407121777 | 0.0000 | down |
|  | Quinolines and derivatives | Kynurenic acid | 1.499914528 | 0.0000 | down |
|  |  | Quinoline-4-carboxylic acid | 1.448339151 | 0.0000 | down |
|  |  | Xanthurenic Acid | 1.139145818 | 0.0008 | down |
| Phenylpropanoids and polyketides | Flavonoids | Pelargonidin | 1.349013358 | 0.0000 | down |
|  | Isoflavonoids | Daidzein | 1.101694521 | 0.0003 | down |
|  |  | (R)-Equol | 1.425593822 | 0.0000 | down |
|  |  | Genistein 4'-O-glucuronide | 1.279623698 | 0.0001 | down |
|  | Phenylpropanoic acids | 3-Phenyllactic acid | 1.224990228 | 0.0000 | down |
|  |  | 2-Phenylpropionic acid | 1.053556309 | 0.0004 | down |
|  |  | 3-(3,4-dihydroxyphenyl)propanoic acid | 1.078664551 | 0.0006 | up |
| - | - | methadone-d9 | 1.42378843 | 0.0000 | up |
|  |  | D-(-)-Glutamine | 1.417837128 | 0.0000 | up |
|  |  | LPC 20:4 | 1.393040271 | 0.0000 | up |
|  |  | OxPC (16:0-18:0+1O(1Cyc)) | 1.393750743 | 0.0000 | up |
|  |  | Methyl 3-indolyacetate | 1.507025384 | 0.0000 | down |
|  |  | P-Acetamidophenyl-b-D-glucuronide | 1.355301006 | 0.0000 | down |
|  |  | LPE 20:4 | 1.485972979 | 0.0000 | up |
|  |  | Calcium D-Panthotenate | 1.347082347 | 0.0000 | up |
|  |  | 4-Hydroxybutyric acid (GHB) | 1.375901891 | 0.0000 | up |
|  |  | SM (d14:2/20:0) | 1.339239677 | 0.0000 | up |
|  |  | LPC 16:0 | 1.304559903 | 0.0000 | up |
|  |  | trans-Petroselinic Acid | 1.369712616 | 0.0000 | up |
|  |  | LPA 16:0 | 1.3369003 | 0.0000 | up |
|  |  | D-(+)-Glucose | 1.28989909 | 0.0000 | up |
|  |  | LPC 20:0 | 1.285983335 | 0.0000 | down |
|  |  | (±)13-HODE | 1.325870044 | 0.0000 | up |
|  |  | DL-4-Hydroxyphenyllactic acid | 1.286663139 | 0.0000 | up |
|  |  | LysoPE 18:0 | 1.274324675 | 0.0000 | up |
|  |  | Glycerophospho-N-palmitoyl ethanolamine | 1.399409922 | 0.0000 | up |
|  |  | Asp-Phe methyl ester | 1.268382458 | 0.0000 | up |
|  |  | PC (16:0/22:6) | 1.320131052 | 0.0000 | up |
|  |  | FAHFA (18:2/20:4) | 1.3113716 | 0.0000 | up |
|  |  | Taurochenodeoxycholic Acid (sodium salt) | 1.297925618 | 0.0000 | up |
|  |  | 13(S)-HOTrE | 1.26408541 | 0.0000 | up |
|  |  | Glycodeoxycholic Acid (hydrate) | 1.273305424 | 0.0000 | down |
|  |  | 3-amino-1H-pyrazolo[4,3-c]pyridine-4,6-diol | 1.273428367 | 0.0000 | up |
|  |  | Salvinorin A | 1.244196022 | 0.0000 | up |
|  |  | 11(E)-Eicosenoic Acid | 1.34174994 | 0.0000 | up |
|  |  | LPE 16:0 | 1.329006031 | 0.0000 | up |
|  |  | PE (16:0/22:6) | 1.270415823 | 0.0000 | up |
|  |  | Dichloroacetic acid | 1.207912487 | 0.0000 | up |
|  |  | Lysopc 18:1 | 1.180584366 | 0.0000 | down |
|  |  | FAHFA (18:1/20:3) | 1.234692239 | 0.0000 | up |
|  |  | Kynurenic acid O-hexside | 1.163011631 | 0.0000 | down |
|  |  | N-Acetyl-Dl-glutamic acid | 1.178421236 | 0.0000 | down |
|  |  | LPC 19:1 | 1.139127533 | 0.0000 | down |
|  |  | 2-{2-[2,5-di(methoxycarbonyl)anilino]-2-oxoethoxy}acetic acid | 1.137195836 | 0.0000 | down |
|  |  | 7-Hydroxy-4-chromone | 1.148233623 | 0.0000 | down |
|  |  | gamma-Nonanolactone | 1.130551039 | 0.0000 | up |
|  |  | alpha-Benzylsuccinic acid | 1.214395657 | 0.0000 | down |
|  |  | 3-anilino-5-(4-chlorophenyl)cyclohex-2-en-1-one | 1.140223619 | 0.0000 | up |
|  |  | 2-{2-oxo-2-[(2-oxo-3-azepanyl)amino]ethoxy}acetic acid | 1.184089703 | 0.0000 | down |
|  |  | 19(R)-hydroxy Prostaglandin A2 | 1.167690669 | 0.0000 | down |
|  |  | 12-oxo Phytodienoic Acid | 1.163487083 | 0.0000 | up |
|  |  | FAHFA (18:0/20:2) | 1.151575514 | 0.0000 | up |
|  |  | LPC 20:1 | 1.150623999 | 0.0000 | down |
|  |  | 4-oxo-4-(4-toluidino)but-2-enoic acid | 1.125219597 | 0.0000 | up |
|  |  | PC (18:2/20:4) | 1.068436246 | 0.0000 | up |
|  |  | 4-(octyloxy)benzoic acid | 1.050483199 | 0.0001 | down |
|  |  | 11(Z),14(Z)-Eicosadienoic Acid | 1.104590873 | 0.0001 | up |
|  |  | Guanosine monophosphate (GMP) | 1.112074586 | 0.0001 | up |
|  |  | FAHFA (16:0/18:2) | 1.095303871 | 0.0001 | up |
|  |  | 3-(2-Naphthyl)-D-Alanine | 1.06239133 | 0.0001 | down |
|  |  | NSI-189 | 1.01454315 | 0.0002 | down |
|  |  | LPE 22:6 | 1.272307431 | 0.0002 | up |
|  |  | Lysopc 14:0 | 1.017824839 | 0.0002 | down |
|  |  | 3,4-dimethoxy-N-[3-(1,3,4-oxadiazol-2-yl)phenyl]benzenesulfonamide | 1.103269107 | 0.0002 | down |
|  |  | 5-Methoxyindole-3-Carbaldehyde | 1.064104193 | 0.0002 | up |
|  |  | FAHFA (22:4/18:0) | 1.007340919 | 0.0003 | up |
|  |  | MAG (18:2) | 1.012350102 | 0.0003 | up |
|  |  | N1-[4-(cyanomethyl)phenyl]-4-chlorobenzamide | 1.107408942 | 0.0005 | down |
|  |  | LPC 20:2 | 1.11806922 | 0.0005 | down |
|  |  | Glycochenodeoxycholic Acid (sodium salt) | 1.065225135 | 0.0007 | down |
|  |  | Lysopc 16:1 | 1.022263652 | 0.0008 | down |
|  |  | 6-Methoxy-2-naphthoic acid | 1.081461537 | 0.0008 | down |
|  |  | LPC 17:1 | 1.013022259 | 0.0015 | down |
|  |  | N-METHYL (-)EPHEDRINE | 1.045336062 | 0.0026 | down |
|  |  | DL-m-Tyrosine | 1.657427188 | 0.0000 | down |
|  |  | indoline-2-carboxylic acid | 1.624294257 | 0.0000 | down |
|  |  | 1-(3-methoxy-2-nitrostyryl)pyrrolidine | 1.614221282 | 0.0000 | down |
|  |  | 6-Deoxy-D-glucose | 1.673757984 | 0.0000 | up |
|  |  | α-Methyl-DL-histidine | 1.622882284 | 0.0000 | up |
|  |  | PC (16:2e/4:0) | 1.734243581 | 0.0000 | down |
|  |  | 7-Hydroxy-3,4-dihydrocarbostyril | 1.623251133 | 0.0000 | down |
|  |  | LPC 15:1 | 1.573714323 | 0.0000 | down |
|  |  | PC (16:1e/3:0) | 1.505824373 | 0.0000 | down |
|  |  | 8-Hydroxyquinoline | 1.644680962 | 0.0000 | down |
|  |  | ACar 20:5 | 1.485118797 | 0.0000 | up |
|  |  | benzaldehyde 1-(2,4-dinitrophenyl)hydrazone | 1.598799731 | 0.0000 | down |
|  |  | ACar 17:0 | 1.467730843 | 0.0000 | up |
|  |  | ACar 18:0 | 1.532105899 | 0.0000 | up |
|  |  | Indoxyl-β-D-glucuronide | 1.460727727 | 0.0000 | down |
|  |  | 5-(2,5-dihydroxyhexyl)oxolan-2-one | 1.473436726 | 0.0000 | up |
|  |  | PC (16:0e/3:0) | 1.441909994 | 0.0000 | down |
|  |  | N-(5-acetamidopentyl)acetamide | 1.461816557 | 0.0000 | down |
|  |  | 5-Methyl-dl-tryptophan | 1.445603289 | 0.0000 | down |
|  |  | 2-methyl-6-{[(5-phenyl-2-thienyl)carbonyl]amino}benzoic acid | 1.511292564 | 0.0000 | down |
|  |  | ACar 20:4 | 1.454644159 | 0.0000 | up |
|  |  | N1,N1-diethyl-4-[2-(4-bromophenyl)diaz-1-enyl]aniline | 1.448775736 | 0.0000 | down |
|  |  | LPC 15:0 | 1.532040338 | 0.0000 | down |
|  |  | LPC 16:2 | 1.431949958 | 0.0000 | down |
|  |  | PC (14:1e/4:0) | 1.426729217 | 0.0000 | down |
|  |  | 1,3-dioxo-2,3-dihydro-1H-benzo[de]isoquinolin-2-yl acetate | 1.439229545 | 0.0000 | down |
|  |  | methyl isoquinoline-3-carboxylate | 1.377865823 | 0.0000 | up |
|  |  | ACar 10:1 | 1.44349683 | 0.0000 | up |
|  |  | Pyridoxine O-Glucoside | 1.386594264 | 0.0000 | down |
|  |  | Lysine Butyrate | 1.412557493 | 0.0000 | down |
|  |  | PC (14:1e/3:0) | 1.422070151 | 0.0000 | down |
|  |  | 3-(propan-2-yl)-octahydropyrrolo[1,2-a]pyrazine-1,4-dione | 1.430589151 | 0.0000 | down |
|  |  | PC (14:1e/8:0) | 1.513971134 | 0.0000 | down |
|  |  | Cyclohexyl fentanyl-d5 | 1.350763816 | 0.0000 | up |
|  |  | PC (14:0e/3:0) | 1.434052409 | 0.0000 | down |
|  |  | ACar 18:2 | 1.406075147 | 0.0000 | up |
|  |  | (±)-Abscisic acid | 1.367552453 | 0.0000 | down |
|  |  | Prostaglandin A3 | 1.402350523 | 0.0000 | down |
|  |  | 3-(4-methoxyphenyl)-1-methyl-1H-1,2,4-triazol-5-ol | 1.38232628 | 0.0000 | down |
|  |  | PC (16:2e/2:0) | 1.352687142 | 0.0000 | down |
|  |  | Dl-2-Amino-3-phosphonopropionic acid | 1.321628762 | 0.0000 | down |
|  |  | (2S)-2-(2-hydroxypropan-2-yl)-2H,3H,7H-furo[3,2-g]chromen-7-one | 1.38238556 | 0.0000 | down |
|  |  | ethyl 4-hydroxy-2-[(4-methoxyphenoxy)methyl]pyrimidine-5-carboxylate | 1.328353257 | 0.0000 | down |
|  |  | Mag (18:1) | 1.361361498 | 0.0000 | down |
|  |  | 2-cyano-3-(3,4-dimethoxyphenyl)acrylic acid | 1.394024444 | 0.0000 | down |
|  |  | N'-hydroxy-2,5-dimethoxybenzenecarboximidamide | 1.333373774 | 0.0000 | down |
|  |  | PC (14:1e/6:0) | 1.45272733 | 0.0000 | down |
|  |  | 6-Methoxyquinoline N-oxide | 1.312254248 | 0.0000 | up |
|  |  | 3'-Adenosine monophosphate (3'-AMP) | 1.357271028 | 0.0000 | down |
|  |  | ACar 18:1 | 1.362954026 | 0.0000 | up |
|  |  | bicyclo[2.2.2]oct-2-en-1-yl 4-methylbenzene-1-sulfonate | 1.315520708 | 0.0000 | up |
|  |  | ACar 15:0 | 1.279230858 | 0.0000 | up |
|  |  | 3-[3-(beta-D-Glucopyranosyloxy)-2-hydroxyphenyl]propanoic acid | 1.265928409 | 0.0000 | down |
|  |  | DL-Lysine | 1.251513345 | 0.0000 | up |
|  |  | L-beta-Imidazolelactic acid | 1.27884759 | 0.0000 | down |
|  |  | PC (19:2/18:4) | 1.378785395 | 0.0000 | down |
|  |  | 4-(4-methoxyphenyl)-2-(methylthio)pyrimidine | 1.353662579 | 0.0000 | down |
|  |  | 5,7-dihydroxy-2-phenyl-4H-chromen-4-one | 1.355837571 | 0.0000 | down |
|  |  | Prostaglandin K2 | 1.230823155 | 0.0000 | down |
|  |  | 1,7-bis(3,4-dihydroxyphenyl)heptan-3-one | 1.364257124 | 0.0000 | down |
|  |  | 3-(2-methylpropyl)-octahydropyrrolo[1,2-a]pyrazine-1,4-dione | 1.614181827 | 0.0000 | down |
|  |  | 11-dehydro Thromboxane B2 | 1.23791624 | 0.0000 | down |
|  |  | FRH | 1.260032553 | 0.0001 | down |
|  |  | 13,14-dihydro-15-keto Prostaglandin A2 | 1.226748125 | 0.0001 | down |
|  |  | LPC 18:3 | 1.265558528 | 0.0001 | down |
|  |  | ACar 20:0 | 1.280281985 | 0.0001 | up |
|  |  | PC (16:2e/6:0) | 1.297307402 | 0.0001 | down |
|  |  | Boc-beta-cyano-L-alanine | 1.302463958 | 0.0001 | down |
|  |  | PC (22:5e/16:0) | 1.239425655 | 0.0001 | up |
|  |  | 2-({[2-(1H-benzimidazol-2-yl)phenyl]imino}methyl)-4,6-dichlorophenol | 1.343073403 | 0.0001 | down |
|  |  | L-Glutathione (reduced) | 1.425240351 | 0.0001 | down |
|  |  | ACar 20:1 | 1.294225008 | 0.0001 | up |
|  |  | PC (18:5e/4:0) | 1.158219971 | 0.0001 | down |
|  |  | PC (17:1/17:2) | 1.26756247 | 0.0001 | down |
|  |  | ACar 20:2 | 1.293503174 | 0.0002 | up |
|  |  | ACar 13:0 | 1.276080918 | 0.0002 | up |
|  |  | 4-(allyloxy)-1,2-dihydroquinolin-2-one | 1.160019539 | 0.0002 | down |
|  |  | ACar 18:3 | 1.229217886 | 0.0002 | up |
|  |  | 2-(acetylamino)-3-(1H-indol-3-yl)propanoic acid | 1.250097363 | 0.0002 | down |
|  |  | o-Veratraldehyde | 1.14264714 | 0.0002 | down |
|  |  | ACar 16:1 | 1.14338693 | 0.0003 | up |
|  |  | 2-(cyclopropylcarbonyl)-3-(4-fluoroanilino)acrylonitrile | 1.236170145 | 0.0003 | down |
|  |  | 5α-Pregnan-3,20-dione | 1.259709014 | 0.0003 | down |
|  |  | EPK | 1.122841823 | 0.0003 | up |
|  |  | 1,2-dihydroxyheptadec-16-yn-4-yl acetate | 1.259897898 | 0.0003 | down |
|  |  | Di(2-ethylhexyl) phthalate | 1.125384033 | 0.0004 | down |
|  |  | 5-(6-hydroxy-6-methyloctyl)-2,5-dihydrofuran-2-one | 1.258073532 | 0.0004 | down |
|  |  | Ne-(1-Carboxymethyl)-L-lysine | 1.221412867 | 0.0004 | down |
|  |  | H-Gly-Pro-OH | 1.134237113 | 0.0006 | down |
|  |  | 2-[5-(2-hydroxypropyl)oxolan-2-yl]propanoic acid | 1.064997937 | 0.0009 | down |
|  |  | ACar 24:0 | 1.107051429 | 0.0011 | up |
|  |  | (5-L-Glutamyl)-L-Amino Acid | 1.090455931 | 0.0011 | down |
|  |  | SM (d14:2/27:0) | 1.01651851 | 0.0011 | up |
|  |  | Dehydrocholic acid | 1.059982312 | 0.0011 | up |
|  |  | LSD-d3 | 1.085493962 | 0.0014 | up |
|  |  | 6-(7-methyloctyl)-1H,3H,4H,6H-furo[3,4-c]furan-1-one | 1.145746967 | 0.0015 | down |
|  |  | N1-[2-oxo-6-(1H-pyrrol-1-yl)-2H-chromen-3-yl]acetamide | 1.187245932 | 0.0020 | down |
|  |  | SM (d14:2/14:0) | 1.189300508 | 0.0027 | down |
|  |  | MAG (18:3) | 1.036664955 | 0.0028 | down |
|  |  | PC (18:3/18:3) | 1.187150267 | 0.0030 | down |
|  |  | 2-methyl-2,3,4,5-tetrahydro-1,5-benzoxazepin-4-one | 1.036092725 | 0.0032 | down |
|  |  | SM (d14:0/12:1) | 1.175949379 | 0.0034 | down |
|  |  | ACar 24:1 | 1.056261868 | 0.0046 | up |
|  |  | SM (d16:0/12:1) | 1.153394794 | 0.0050 | down |
|  |  | Tetranor-12(S)-HETE | 1.212696422 | 0.0061 | down |
|  |  | SM (d16:0/12:0) | 1.129247983 | 0.0087 | down |
|  |  | 4-[(2-thienylmethylidene)amino]benzoic acid | 1.039192939 | 0.0108 | up |
